# Short-Term Mortality and Its Predictors in Alcohol-Related Acute-on-Chronic Liver Failure: A Systematic Review and Meta-Analysis

**DOI:** 10.3390/healthcare14203543

**Published:** 2026-10-21

**Authors:** Ayauzhan A. Sapanova, Venera S. Rakhmetova, Assylzhan M. Messova, Amin Tamadon, Sagira T. Abdrakhmanova

**Affiliations:** 1 Department of Internal Diseases, Astana Medical University, Astana 010000, Kazakhstan; doctorayauzhan86@gmail.com (A.A.S.); venerakhmetova@gmail.com (V.S.R.); 2 Department of Pediatric Diseases, Astana Medical University, Astana 010000, Kazakhstan; abdrakhmanova.s@amu.kz; 3 Department of Natural Sciences, West Kazakhstan Marat Ospanov Medical University, Aktobe 030019, Kazakhstan; amintamaddon@yahoo.com

**Keywords:** alcohol-related liver disease, acute-on-chronic liver failure, short-term mortality, prognostic factors, organ failure, infection, hepatic encephalopathy, MELD, CLIF-C ACLF, meta-analysis

## Abstract

**Highlights:**

• Main findings: Pooled mortality was 36.0% at 28/30 days and 50.2% at 90 days (11 direct cohorts each), with substantial between-study heterogeneity (95% prediction intervals 17.3–60.3% and 19.2–81.0%, respectively). In a structured qualitative synthesis, mainly of direct alcohol-related ACLF cohorts, organ-failure burden, infection or sepsis, hepatic encephalopathy, renal dysfunction, shock, and higher MELD-, CLIF-, or AARC-based severity were the most consistent adverse signals.
• Implications: Risk assessment should combine a framework-appropriate score with serial evaluation of infection, neurological and circulatory dysfunction, renal and respiratory failure, and short-interval clinical trajectory. Novel biomarkers require external validation.

**Abstract:**

**Background/Objectives:** Alcohol-related acute-on-chronic liver failure (ACLF) carries high early mortality, but prognostic evidence varies by definition, endpoint, and adjustment. We estimated short-term mortality and synthesized clinically useful predictors. **Methods:** PubMed/MEDLINE, Scopus, and Web of Science were searched to 25 June 2026 and, with a revised strategy, to 28 September 2026. Direct alcohol-related ACLF cohorts formed the core evidence base. Mortality was pooled using REML random-effects models with Hartung–Knapp confidence intervals and 95% prediction intervals (PIs); predictors were synthesized qualitatively. Risk of bias and certainty were assessed with QUIPS and GRADE. **Results**: Forty reports (35 independent cohorts) were included. Pooled mortality was 36.0% at 28/30 days (11 cohorts; 95% CI 28.9–43.8; PI 17.3–60.3%) and 50.2% at 90 days (11 cohorts; 95% CI 39.5–60.8; PI 19.2–81.0%), without significant differences between APASL/AARC- and EASL-CLIF-defined cohorts. Qualitatively, organ-failure burden, infection or sepsis, hepatic encephalopathy, renal dysfunction, shock, and higher MELD-, CLIF-, or AARC-based severity were the most consistent adverse signals; pooled predictor effects could not be estimated. **Conclusions:** About one-third of patients die by 28/30 days and half by 90 days, but mortality varies widely across settings. Predominantly very low-certainty predictor evidence supports framework-appropriate scores and repeated assessment rather than a universal cutoff.

## 1. Introduction

Acute-on-chronic liver failure (ACLF) is a distinct syndrome in patients with chronic liver disease or cirrhosis who develop acute deterioration, organ failure, and a high risk of death over weeks to months [1]. International frameworks are not interchangeable: the EASL-CLIF approach emphasizes hepatic and extrahepatic organ failure, whereas APASL/AARC definitions focus on acute hepatic injury, jaundice, coagulopathy, ascites, and hepatic encephalopathy; the World Gastroenterology Organisation additionally classifies patients according to the stage of underlying chronic liver disease [2,3,4]. Alcohol may contribute both as the cause of chronic liver disease and as an acute precipitant through alcohol-associated hepatitis, and patients frequently present with malnutrition, systemic inflammation, immune dysfunction, bacterial infection, and multiorgan failure [5].

ACLF is dynamic. Organ failures may improve or progress during the first days after admission, and prognosis changes accordingly [6]. Infection can be a precipitating event, a baseline complication, or a time-varying event after ACLF onset; each pathway can amplify systemic inflammation and accelerate renal, circulatory, respiratory, and neurological dysfunction [7]. Broader acute-decompensation cohorts similarly show that trajectories, rather than a single admission measurement, distinguish patients with reversible deterioration from those who progress to persistent or worsening ACLF [8].

Alcohol-specific prognostic evidence is dispersed across registries, single-center cohorts, intensive-care populations, severe alcohol-associated hepatitis studies, and biomarker investigations [9,10,11,12,13,14,15,16,17,18,19,20,21,22,23,24,25,26]. These reports use different ACLF definitions, predictor scales, follow-up windows, effect measures, and adjustment sets. Mortality may be reported at 28 or 30 days, at 90 days or three months, during the index hospitalization, or as transplant-free or competing-risk survival. Individual predictors also overlap with diagnostic criteria and composite scores: creatinine is both a marker of renal dysfunction and a component of MELD- and ACLF-based scores, while hepatic encephalopathy may be modeled independently, incorporated into cerebral organ failure, or included in disease grading.

Conventional scores such as MELD summarize hepatic synthetic and renal dysfunction, whereas CLIF-C ACLF incorporates age, inflammation, and the extent of organ failure [27]. In APASL-defined populations, the AARC score provides a framework-specific alternative based on bilirubin, INR, lactate, creatinine, and hepatic encephalopathy [28]. Their relative performance may therefore depend on the diagnostic framework, clinical setting, and mortality window. New biomarkers and study-derived models may improve discrimination, but their incremental value and transportability remain uncertain.

This systematic review and meta-analysis aimed to estimate 28/30-day and 90-day mortality in adults with alcohol-related ACLF and to identify the most reproducible clinical, biochemical, inflammatory, organ-failure, and score-based predictors. Secondary objectives were to compare prognostic-score discrimination, distinguish direct alcohol-related ACLF evidence from supportive or indirect evidence, and examine the influence of endpoint definition, ACLF framework, clinical setting, and risk of bias.

## 2. Materials and Methods

### 2.1. Protocol, Reporting Standards, and Registration

The review was conducted in accordance with PRISMA 2020 [29]. Search reporting followed PRISMA-S [30], and methodological decisions for observational prognostic research were informed by MOOSE and the Cochrane Handbook [31,32]. The protocol was registered in PROSPERO (CRD420261433393). Aggregate data from published reports were used; therefore, institutional review board approval and individual informed consent were not required. Any deviations from the prespecified protocol were documented and justified in the review report. Two deviations from the registered protocol occurred: the search strategy was revised and rerun after the targeted update (Section 2.3), and a framework-stratified analysis was added post hoc in response to peer review (Section 2.6); the PROSPERO record was updated accordingly.

### 2.2. Eligibility Criteria

The core population of interest was adults with ACLF in whom alcohol was the cause of the underlying chronic liver disease, studied either in an alcohol-restricted ACLF cohort or in an extractable alcohol-related ACLF subgroup (direct evidence). Only such studies were eligible for the primary mortality meta-analyses and could support the principal prognostic conclusions. Studies of severe alcohol-associated hepatitis, mixed-etiology ACLF, or combined acute decompensation/ACLF populations without a separable alcohol-related ACLF analysis were retained only as supportive evidence to corroborate or contextualize direct findings; they did not enter the primary mortality meta-analyses. Eligible studies enrolled adults (aged 18 years or older) with alcohol-related cirrhosis, alcohol-associated liver disease, or alcohol-associated hepatitis complicated by ACLF. ACLF could be defined using EASL-CLIF/CANONIC, APASL/AARC, WGO, CLIF-SOFA, or another clearly described clinical framework. Mixed-etiology studies were eligible only when an alcohol-related subgroup was extractable or when the cohort provided clinically relevant supportive evidence. Studies of severe alcohol-associated hepatitis were eligible when ACLF was present in most participants, an ACLF subgroup was identifiable, or ACLF-specific scores or organ-failure variables were evaluated.

Predictors included demographics and comorbidities; infection, sepsis, systemic inflammatory response, hepatic encephalopathy, shock, acute kidney injury, hepatorenal syndrome, and organ-support requirements; biochemical and inflammatory markers; organ-failure number or grade; conventional liver-specific scores; ACLF-specific scores; and novel biomarkers or study-derived models. Comparators included survivors versus nonsurvivors, patients with versus without a predictor, higher versus lower predictor categories, different ACLF grades, continuous predictor increments, or alternative prognostic models.

The primary outcomes were all-cause mortality at 28 or 30 days and at 90 days. Three-month mortality was considered clinically adjacent to 90-day mortality only in sensitivity analysis. In-hospital mortality was analyzed separately because follow-up duration depended on length of stay and discharge practice. Long-term outcomes without a fixed short-term window, transplant-free or competing-risk outcomes that could not be reconciled with conventional mortality, and composite endpoints in which death could not be separated were retained only for narrative interpretation.

Eligible designs were prospective or retrospective cohort studies, registry analyses, prognostic-factor studies, and prognostic-model development or validation studies. Reviews, editorials, case reports, conference abstracts without adequate data, nonclinical studies, exclusively post-transplant studies, and reports without extractable alcohol-related ACLF or short-term outcome data were excluded.

### 2.3. Information Sources, Search, and Study Selection

**Figure 1:**
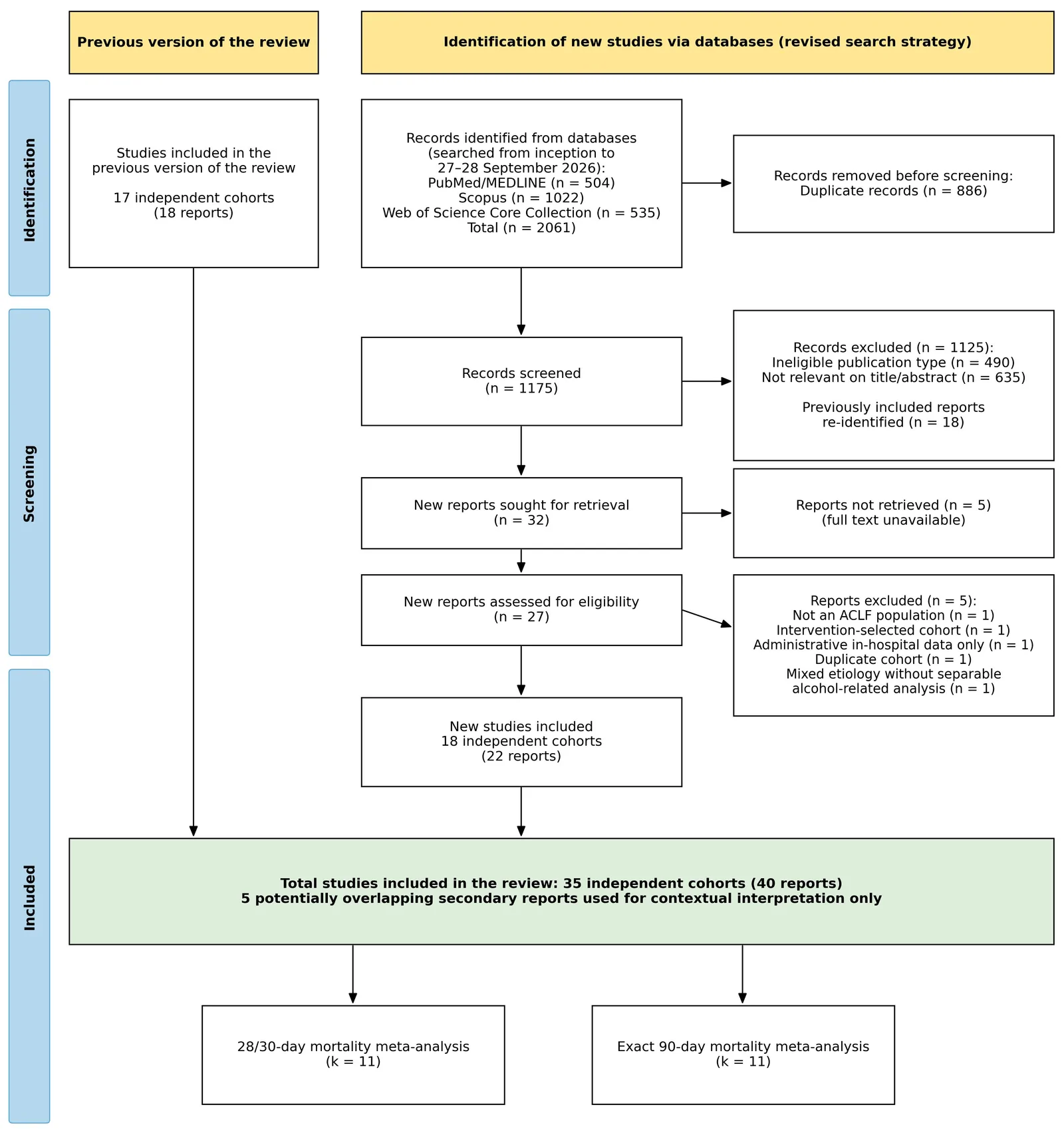
PRISMA 2020 flow diagram for the updated review. Studies included in the previous version (17 independent cohorts; 18 reports, including two identified by a targeted manual search) are shown on the left. The revised database search (27–28 September 2026) retrieved 2061 records and yielded 22 additional eligible reports representing 18 independent cohorts. In total, 40 reports representing 35 independent cohorts were included; five potentially overlapping secondary reports were used only for contextual interpretation. The primary meta-analyses included 11 cohorts for 28/30-day and 11 for exact 90-day mortality.

The original search of PubMed/MEDLINE, Scopus, and Web of Science Core Collection covered the period from database inception to 25 June 2026; the final search, using a revised strategy, was run from inception to 27–28 September 2026 (Supplementary Table S1). Records were imported into EndNote 20.6.0 (Clarivate, Philadelphia, PA, USA) reference-management software and deduplicated. Two reviewers independently screened titles and abstracts and then assessed potentially eligible full texts. Disagreements were resolved by discussion and re-examination of the source report, with third-reviewer adjudication when required. Reasons for full-text exclusion were recorded. During final evidence reconciliation, before the quantitative analyses were finalized, a targeted evidence update was performed to identify eligible reports not retrieved by the original database searches. The update consisted of a manual search of recently published literature. Records identified by the update were screened and assessed by the same two reviewers using the eligibility criteria and procedures applied to the original search. This process identified two additional full-text reports, Saini et al. [25] and Glisic et al. [26], both of which met the eligibility criteria; both reports were identified through this manual search. No other new eligible reports were identified by the targeted update. Because the identification of these reports suggested that the original strategy may have been overly restrictive, the search strategy was then systematically revised by replacing the phrase-based alcohol block with truncated terms (alcohol*, ethanol, ALD, ArLD, AALD) and the Medical Subject Headings “Liver Diseases, Alcoholic” and “Alcoholism”, adding ACLF synonyms and ACLF-specific scores (CLIF-C, CLIF-SOFA, CLIF-OF, CANONIC, AARC score), broadening the outcome block with truncated terms, and removing the study-design block and the transplantation exclusion, and was rerun in PubMed/MEDLINE, Scopus, and Web of Science from inception to 27–28 September 2026; the revised strategies are reported in Supplementary Table S1. Records were deduplicated and screened using the same dual-reviewer procedure: 2061 records were retrieved (PubMed/MEDLINE 504, Scopus 1022, Web of Science 535); after removal of 886 duplicates, 1175 records were screened, including all 18 previously included reports. Thirty-two new reports were sought for full-text assessment, of which five could not be retrieved; of the 27 reports assessed, 22 met the eligibility criteria, representing 18 additional independent cohorts (Figure 1). The revised strategy retrieved all 18 previously included reports, including Saini et al. [25] and Glisic et al. [26]. Their omission from the original search was attributable to three features of the original strategy: the alcohol block used disease-specific phrases (e.g., “alcohol-related liver disease”) that did not capture reports describing “alcohol-related acute-on-chronic liver failure”; a study-design block required design terms in the title or abstract; and a NOT operator excluded any record mentioning transplantation. Potentially overlapping reports were linked at the cohort level using study center, investigators, recruitment context, eligibility criteria, and sample characteristics. When participant independence could not be established, the broader cohort report was retained as the independent synthesis unit, whereas the additional report was used only for narrative contextualization to avoid double counting.

### 2.4. Data Extraction and Predictor Classification

A standardized extraction form was piloted before use. One reviewer extracted study characteristics, population and ACLF definitions, sample size, mortality events, follow-up, transplantation and censoring procedures, predictor definitions and timing, crude and adjusted effect estimates, covariates, and discrimination metrics; a second reviewer verified all entries against the source reports. Adjusted estimates from the most comprehensive clinically appropriate model were preferred. Univariable estimates were retained only when no adjusted estimate was available and were clearly labeled. When the number of deaths was not reported directly, it was derived from reported Kaplan–Meier or cumulative-incidence estimates and the cohort size, or by subtraction; such derived counts were identified and examined in sensitivity analyses.

Evidence was classified as direct when a study was restricted to alcohol-related ACLF or provided an extractable alcohol-related ACLF subgroup. Supportive or indirect evidence included severe alcohol-associated hepatitis, mixed-etiology ACLF, and combined acute decompensation/ACLF populations without a fully separable alcohol-related ACLF analysis. Jindal et al. [11] was classified as near-direct because most participants in this severe alcohol-associated hepatitis cohort had ACLF. To keep this distinction visible throughout, the evidence type is summarized for each study in the “Evidence type” column of the study characteristics table (Section 3.1), labeled for each predictor estimate in predictor tables (Section 3.3), and stated in the figurelegends . Predictors were grouped into clinical, biochemical, inflammatory/immune, organ-failure/severity, established-score, and novel-biomarker domains.

### 2.5. Risk of Bias

Two reviewers independently assessed prognostic-factor studies using the Quality In Prognosis Studies (QUIPS) tool across study participation, attrition, prognostic-factor measurement, outcome measurement, confounding, and statistical analysis/reporting [33]. Because the review focused on prognostic factors rather than formal validation of complete prediction models, QUIPS was applied consistently; model-development and score-performance evidence was treated as supportive and interpreted with attention to PROBAST principles [34]. Overall risk was classified as high when at least one domain was high or at least three domains were moderate; moderate when no domain was high and one or two were moderate; and low when all domains were low. Judgments informed interpretation rather than being converted to a numerical quality score.

### 2.6. Statistical Analysis

A narrative synthesis was performed for all eligible studies. Quantitative pooling was restricted to studies that were clinically comparable in population, endpoint, and effect definition. The primary early-mortality model combined clinically adjacent 28- and 30-day endpoints; a strict 28-day analysis excluded 30-day reports. The primary later model included exact 90-day endpoints; three-month reports were added only in sensitivity analysis. In-hospital mortality was kept separate.

Mortality proportions were transformed to the logit scale and pooled with restricted maximum-likelihood random-effects models [35]. Hartung–Knapp confidence intervals and 95% prediction intervals were calculated, and analyses were conducted in Rversion 4.5.2 (R Foundation for Statistical Computing, Vienna, Austria; https://www.R-project.org/) via RStudio 2025.09.2 (Posit Software, PBC, Boston, MA, USA) using the metafor package version 5.0.1 [36,37]. Heterogeneity was described using Cochran Q, I^2^, and tau^2^ and interpreted together with clinical diversity and the number of studies [38].

Hazard ratios (HRs), odds ratios (ORs), subdistribution HRs, and time ratios were not treated as interchangeable. Continuous estimates were retained in their reported units, and predictor effects based on incompatible cutoffs or transformations were not combined. AUROC and C-index values were displayed on a common discrimination scale for descriptive comparison but were not pooled. Predictor-specific pooling was undertaken only when independent studies used sufficiently comparable definitions, effect measures, populations, and mortality windows. Because this condition was not met for any individual predictor, prognostic factors were synthesized using a structured qualitative approach that considered the direction, consistency, adjustment, directness (direct vs. supportive evidence), and precision of the reported associations; no pooled predictor effect estimates were generated.

Prespecified sensitivity analyses used stricter or broader endpoint and eligibility definitions. The strict early analysis excluded 30-day reports, whereas broader analyses added clinically restricted or methodologically less direct cohorts and a clearly defined three-month cohort. ICU in-hospital mortality was retained as a separate descriptive analysis, and potentially overlapping reports were excluded from independent counts and pooling. Formal small-study-effect testing was planned only for syntheses with at least 10 independent studies; because all principal models included fewer than 10, funnel plots and Egger regression were not used to infer publication bias [39]. In response to peer review, a post hoc analysis stratified the primary mortality analyses by ACLF diagnostic framework (APASL/AARC, EASL-CLIF, WGO, or Chinese national criteria). Strata containing at least two studies were pooled with the same model, and single-study strata were reported descriptively. Differences between APASL/AARC- and EASL-CLIF-defined cohorts were tested with a mixed-effects meta-regression using the Hartung–Knapp adjustment; a cohort reporting separate APASL-only and EASL-CLIF subgroups contributed each subgroup to its respective stratum. Additional sensitivity analyses excluded the cohort with high attrition and cohorts with derived death counts.

### 2.7. Certainty of Evidence

Certainty for the major prognostic-factor domains was evaluated using an adapted GRADE framework for prognostic-factor research [40,41]. Judgments considered risk of bias, inconsistency, indirectness, imprecision, publication bias, magnitude and consistency of association, dose–response, and the likelihood that residual confounding explained the observed association. Direct and supportive evidence were considered separately, and certainty was rated high, moderate, low, or very low by two reviewers with consensus resolution.

## 3. Results

### 3.1. Study Selection and Characteristics

The original database searches identified 270 records: 128 from PubMed/MEDLINE, 99 from Scopus, and 43 from Web of Science. After removal of 86 duplicates, 184 records were screened and 156 were excluded by title and abstract. All 28 reports sought for full-text review were obtained; 12 were excluded for lack of alcohol-specific ACLF data, a relevant short-term mortality outcome, extractable prognostic data, or an eligible design, leaving 16 eligible reports. A targeted manual search (Section 2.3) identified two further eligible reports, Saini et al. [25] and Glisic et al. [26]. The revised comprehensive search retrieved 2061 records; after removal of 886 duplicates, 1175 records were screened, including all 18 previously included reports. Of 32 new reports sought for full-text assessment, five could not be retrieved and five of the 27 assessed were excluded (Supplementary Table S2), leaving 22 additional eligible reports [42,43,44,45,46,47,48,49,50,51,52,53,54,55,56,57,58,59,60,61,62,63]. In total, 40 reports representing 35 independent cohorts were included (Figure 1). Five reports were treated as potentially overlapping secondary reports and were used only for contextual interpretation: Glisic et al. [26] (same center and investigators as Matovic Zaric et al. [16]); Maiwall et al. [52], Kumar et al. [50], and Thanapirom et al. [61] (APASL ACLF Research Consortium registry, overlapping with Duseja et al. [9]); and Shalimar et al. [59] (same center and period as Sonika et al. [60]).

The 35 independent cohorts were published between 2010 and 2026. Twenty-two were retrospective and 13 prospective or prospectively observed. Twenty-one were conducted in Asia or the Asia-Pacific region, ten in Europe, and four in the Americas. Full cohorts ranged from 25 to 1216 participants, and alcohol-related ACLF subgroups ranged from 19 to 1216. Populations were predominantly male and middle-aged.

Sixteen cohorts used EASL-CLIF/CANONIC-based criteria, ten used APASL/AARC criteria, one reported both APASL-only and EASL-CLIF subgroups, one used WGO categories, two used Chinese national liver failure criteria, two used Japanese criteria including extended or probable cases, two used study-specific or earlier working definitions, and one evaluated alcohol-associated hepatitis with CLIF-based scores without a separately enumerated ACLF subgroup. Mortality windows included 28 or 30 days, 90 days or three months, and in-hospital death. Transplantation and censoring procedures were inconsistently reported. Table 1 summarizes the design, population, endpoint, evidence type, and contribution of each independent cohort, grouped by role in the synthesis.

**Table 1:** Characteristics and analytical role of the included studies.

Study	Setting/Design	Population and ACLF Framework	Endpoint(s)	Evidence Type	Contribution to Synthesis
A. Direct alcohol-related ACLF cohorts contributing to the primary mortality meta-analyses					
Broekhoven et al., 2024 [42]	Belgium; single-center prospective cohort, 2008–2021	73 ACLF within 184 biopsy-proven alcohol-associated hepatitis; EASL-CLIF	28 and 90 days (KM, LT censored)	Direct (subgroup)	28/30 d: 15/73 *; 90 d: 30/73 *
Castaño-Jiménez et al., 2025 [43]	Mexico; single-center cross-sectional cohort, 2022–2023	33 alcohol-related ACLF; EASL-CLIF	28 and 90 days	Direct	28/30 d: 13/33; 90 d: 20/33; microbiota markers (narrative)
Duseja et al., 2021 [9]	Asia-Pacific AARC registry; retrospective analysis	1216 alcohol-related ACLF; APASL/AARC [3,28]	30 and 90 days	Direct	28/30 d: 392/1216; 90 d: 528/1216; score predictors
Giri et al., 2024 [46]	India; single-center prospective cohort of first decompensation, 2020–2021	60 ACLF within 227 alcohol-related cirrhosis; APASL	30 and 90 days	Direct (subgroup)	28/30 d: 24/60; 90 d: 29/60; creatinine; LaFAS/MELD AUROC
Jindal et al., 2024 [11]	India; retrospective severe alcohol-associated hepatitis cohort	775/819 with ACLF; APASL	90 days	Direct mortality; near-direct predictors	90 d: 223/775; hepatic encephalopathy
Lee et al., 2015 [51]	South Korea; single-center retrospective cohort, 2004–2013	167 ACLF within 345 acutely decompensated alcohol-related cirrhosis; EASL-CLIF	4 weeks	Direct (subgroup)	28/30 d: 68/167; ACLF-grade gradient
Matovic Zaric et al., 2024 [16]	Serbia; prospective hospitalized cohort	38 ACLF within 145 ALD cirrhosis; modified EASL-CLIF	28 days	Direct mortality; supportive predictors	28/30 d: 22/38; LIV-IN discrimination
Ray et al., 2019 [57]	India; single-center cohort with prospective follow-up	133 ACLF within 395 alcohol-related liver disease; APASL	30 and 90 days	Direct (subgroup); high attrition	28/30 d: 61/133; 90 d: 101/133 †; organ-failure count, MDF
Saini et al., 2025 [25]	India; prospective single-center cohort	227 alcohol-related ACLF; APASL/AARC	28 and 90 days	Direct	28/30 d: 109/227; 90 d: 136/227; SIRS, early predictors
Sonika et al., 2018 [60]	India; single-center prospective cohort, 2012–2017	171 alcohol-related ACLF (alcohol as chronic etiology and acute insult); EASL-CLIF	In-hospital, 90 days, 1 year	Direct	90 d: 120/171; HE, creatinine, infection; CLIF-C ACLF/APACHE II
Tong et al., 2022 [21]	China; retrospective single center	100 ALD-ACLF; APASL	28 and 90 days	Direct	28/30 d: 23/100; 90 d: 34/100; GP73, scores
Xi et al., 2018 [62]	China; single-center retrospective cohort, 2005–2017	154 alcohol-related ACLF: 90 APASL-only, 64 EASL-CLIF	28 days	Direct	28/30 d: 48/154 (17/90 APASL-only; 31/64 EASL-CLIF); CLIF-SOFA, MELD
Yao et al., 2026 [63]	China; single-center retrospective cohort, 2015–2024	83 ALD-ACLF; Chinese liver failure criteria	90 days	Direct	90 d: 43/83; WBC, bilirubin, prothrombin time
Zhang et al., 2025 [24]	China; multicenter retrospective cohort	296 alcohol-related ACLF within 955; WGO	28 and 90 days	Direct (subgroup)	28/30 d: 80/296; 90 d: 116/296; CLIF-C ACLF, biomarkers
B. Other direct cohorts (clinically restricted, non-standard definition, other time windows or predictor-only)					
Chen et al., 2016 [44]	China; single-center retrospective cohort, 2008–2015	45 alcohol-related ACLF; Chinese liver failure criteria (2012)	3 months	Direct	Three-month sensitivity (19/45); LAAR, MELD
Igna et al., 2024 [47]	Romania; single-center retrospective ICU cohort, 2022–2023	227 alcohol-related cirrhosis with ACLF; EASL-CLIF	30 days	Direct (ICU-restricted)	Sensitivity (79/227); ACLF-grade gradient; presepsin, procalcitonin
Kamezaki et al., 2026 [48]	Japan; single-center retrospective cohort, 2014–2024	26 alcohol-related cirrhosis with Japanese-defined ACLF (incl. extended)	90 days; 1 year	Direct (non-standard definition)	Sensitivity (≈4/26 †); framework discordance
Katoonizadeh et al., 2010 [49]	Belgium; single-center prospective cohort, 2002–2007	54 alcohol-related ACLF with histologically proven cirrhosis; Jalan–Williams definition	In-hospital; 3 months	Direct	In-hospital 25/54 (descriptive); three-month sensitivity; SIRS, age
Kim et al., 2016 (JGH) [13]	South Korea; retrospective single center	100 alcohol-related ACLF; EASL-CLIF	28 and 90 days	Direct	ACLF grade, infection, score discrimination
Kulkarni et al., 2021 [15]	India; prospective pilot	25 alcohol-induced ACLF; APASL	90 days	Direct (small)	Broader 90-day sensitivity; A-FABP
Mehta et al., 2015 [17]	United Kingdom; prospective hemodynamic study	19 ACLF grades 1–2 within 60 alcohol-related cirrhosis	3 months	Direct (exploratory)	Three-month sensitivity; hemodynamics
Murakami et al., 2023 [53]	Japan; single-center retrospective cohort, 2014–2021	46 alcohol-related ACLF; Japanese criteria incl. extended/probable	28 and 90 days (KM); 6 months	Direct (non-standard definition)	Sensitivity (≈15/46, ≈24/46 †); IL-6, day-4 MELD
Pati et al., 2016 [54]	India; single-center cohort	53 followed-up alcohol-related cirrhosis within 123 ACLF; modified APASL 2009 [64]	3 months	Direct (subgroup); high attrition	Three-month sensitivity (43/53)
Patidar et al., 2022 [55]	USA; two-center retrospective ICU cohort, 2016–2018	83 severe alcohol-associated hepatitis–precipitated ACLF within 463 ACLF; EASL-CLIF	30, 90, 180 days (cumulative incidence)	Direct (subgroup, ICU-restricted)	Sensitivity (≈62/83, ≈68/83 †); ACLF-grade gradient
Peng et al., 2025 [18]	China; retrospective infection-restricted cohort	89 infected ALD-ACLF; APASL	28 days	Direct (clinically restricted)	Broader 28/30-day sensitivity; infection-subgroup model
Slyvka et al., 2018 [20]	Ukraine; retrospective precipitant-defined cohort	67 source-defined ACLF within 150 alcoholic cirrhosis	In-hospital/up to 28 days	Direct (classification concern)	Broader sensitivity/narrative
Vijayalekshmi et al., 2025 [22]	India; prospective mechanistic cohort	25 nonseptic alcohol-related ACLF; APASL	Composite at discharge	Direct	Narrative immune-marker evidence
von Maldeghem et al., 2025 [23]	Germany; retrospective ICU cohort	188 alcohol-related cirrhosis with ACLF; EASL-CLIF	In-hospital	Direct (ICU-restricted)	In-hospital (descriptive); sepsis, ACLF grade
C. Supportive/indirect cohorts (not pooled for mortality)					
Gasperment et al., 2025 [45]	France; nine-ICU retrospective cohort, 2006–2017	187 alcohol-associated hepatitis in ICU, 154 (82%) with ACLF; EASL-CLIF	ICU and in-hospital	Supportive (near-direct)	ICU-mortality predictors (SOFA, lactate, MELD)
Idalsoaga et al., 2024 [10]	Chile; three-center retrospective cohort	92 ACLF within 320 decompensated cirrhosis; mixed etiology, EASL-CLIF	Long-term time to death	Supportive	Grade/trajectory evidence
Kaur et al., 2024 [12]	India; prospective ALD-spectrum cohort	125 ACLF; mortality model combined AD + ACLF	30 days	Supportive	Biomarker model
Kim et al., 2016 (WJG) [14]	South Korea; multicenter retrospective cohort	264 alcohol-associated hepatitis; no separate ACLF denominator	28 and 90 days	Supportive	Score comparison
Perricone et al., 2026 [56]	Italy; single-center retrospective cohort, 2016–2023	100 severe alcohol-associated hepatitis, 43 with ACLF 2–3; EASL-CLIF	Death or LT (composite)	Supportive	ACLF 2–3 and death or LT (narrative)
Rutledge et al., 2024 [58]	USA; single-center retrospective cohort	206 severe alcohol-associated hepatitis declined for LT, 126 (61%) with ACLF; EASL-CLIF	30–180 days	Supportive	ACLF-grade gradient; CLIF-C ACLF discrimination
Sersté et al., 2018 [19]	Belgium; retrospective analysis of prospective severe-AH cohort	79 prevalent ACLF within 165; EASL-CLIF	28 and 168 days	Supportive	Severity gradient; time-varying infection

Note: Studies are grouped by their role in the synthesis and listed alphabetically within groups. The primary meta-analyses included only direct, non-ICU-restricted cohorts using standard ACLF definitions (28/30 days: k = 11; exact 90 days: k = 11). ACLF definitions: APASL [3], AARC [28], EASL-CLIF [1,2], and WGO [4].* Number of deaths derived from the reported Kaplan–Meier estimate and cohort size. † Derived from reported survival or cumulative-incidence estimates, or by subtraction (Ray et al.: 133 minus 32 patients surviving beyond 90 days). Potentially overlapping secondary reports (contextual interpretation only; not counted as independent cohorts or pooled): Glisic et al. [26] (same center as Matovic Zaric et al.); Maiwall et al. [52], Kumar et al. [50], and Thanapirom et al. [61] (APASL ACLF Research Consortium registry, overlapping with Duseja et al.); and Shalimar et al. [59] (same center and period as Sonika et al.). Abbreviations: AARC, APASL ACLF Research Consortium, ACLF, acute-on-chronic liver failure; AD, acute decompensation; A-FABP, adipocyte fatty acid-binding protein; AH, alcohol-associated hepatitis; ALD, alcohol-related liver disease; APACHE II, Acute Physiology and Chronic Health Evaluation II; APASL, Asian Pacific Association for the Study of the Liver; AARC, APASL ACLF Research Consortium; AUROC, area under the receiver operating characteristic curve; CLIF-C ACLF, Chronic Liver Failure Consortium acute-on-chronic liver failure score; CLIF-SOFA, Chronic Liver Failure–Sequential Organ Failure Assessment; d, days; EASL-CLIF, European Association for the Study of the Liver–Chronic Liver Failure Consortium; GP73, Golgi protein 73; HE, hepatic encephalopathy; ICU, intensive care unit; IL-6, interleukin-6; incl., including; JGH, Journal of Gastroenterology and Hepatology; k, number of cohorts; KM, Kaplan–Meier; LAAR, liver-to-abdominal area ratio; LaFAS, lactate-free AARC ACLF score; LIV-IN, Liver Mortality Inpatients prognostic score; LT, liver transplantation; MDF, Maddrey discriminant function; MELD, Model for End-Stage Liver Disease; SIRS, systemic inflammatory response syndrome; SOFA, Sequential Organ Failure Assessment; WBC, white blood cell count; WGO, World Gastroenterology Organisation; WJG, World Journal of Gastroenterology.

### 3.2. Short-Term Mortality

**Figure 2:**
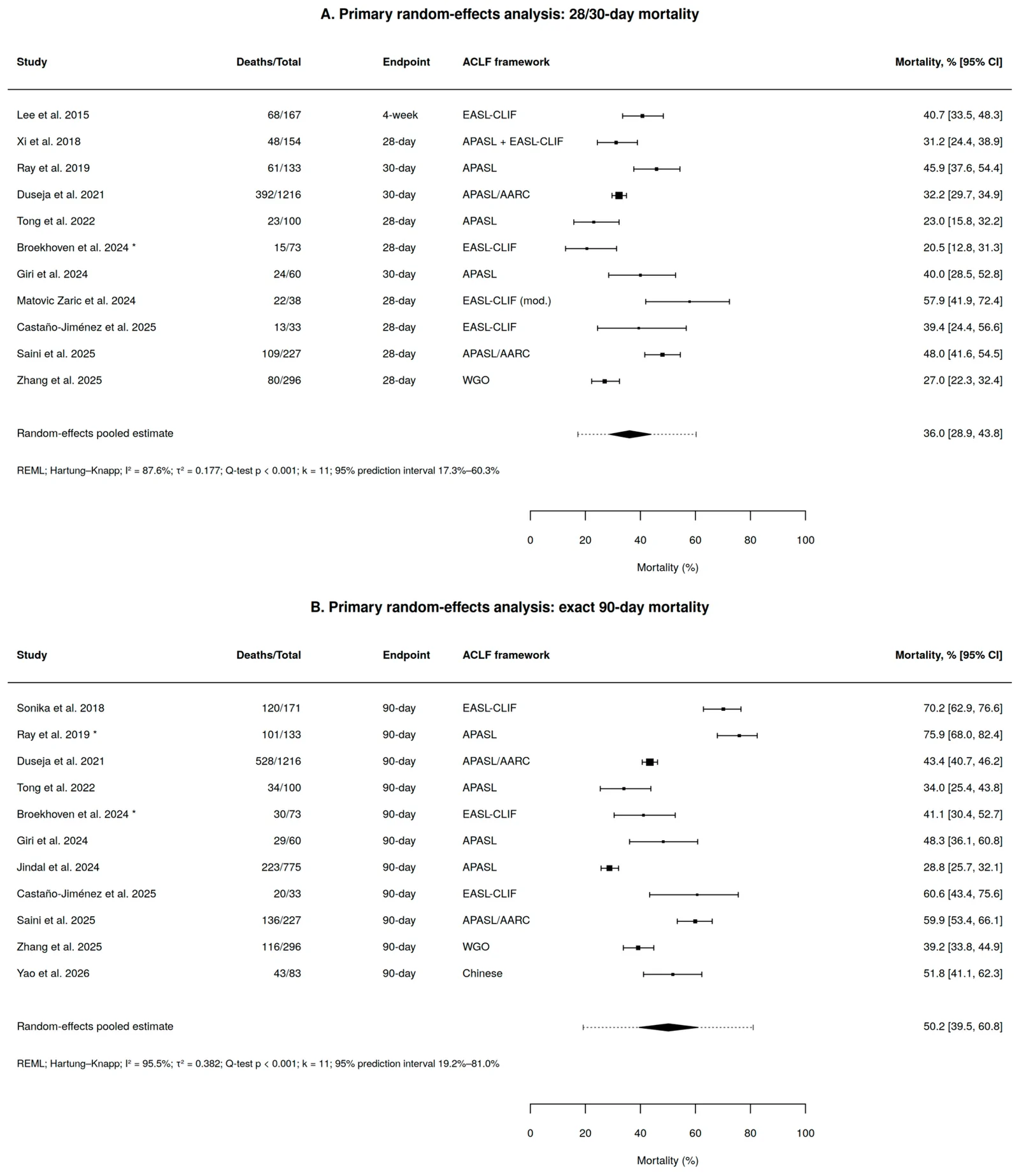
Pooled short-term mortality in alcohol-related ACLF. (**A**) Random-effects meta-analysis of 28/30-day mortality (k = 11) [9,16,21,24,25,42,43,46,51,57,62]. (**B**) Random-effects meta-analysis of exact 90-day mortality (k = 11) [9,11,21,24,25,42,43,46,57,60,63]. Squares show study estimates with 95% confidence intervals; diamonds show pooled estimates; dotted lines show 95% prediction intervals. REML models were fitted on the logit scale with Hartung–Knapp confidence intervals. Both primary analyses included only direct, non-ICU-restricted cohorts using standard ACLF definitions (Table 1); clinically restricted, ICU-restricted, non-standard-definition, and three-month cohorts were added only in the broader sensitivity analyses (Table 2). The wide prediction intervals indicate the range within which mortality in a new comparable setting would be expected to fall. * Number of deaths derived from reported Kaplan–Meier estimates (Broekhoven et al.) [42] or by subtraction (Ray et al. [57], 90 days).

Eleven direct cohorts contributed to the primary 28/30-day analysis (2497 participants; 855 deaths). Study-specific mortality ranged from 20.5% to 57.9%, and the pooled proportion was 36.0% (95% CI 28.9–43.8). Heterogeneity was substantial (I^2^ = 87.6%; τ^2^ = 0.177; Q *p* < 0.001), and the 95% prediction interval was 17.3–60.3% (Figure 2A). The strict 28-day analysis (eight cohorts) yielded 34.9% (95% CI 25.1–46.1; prediction interval 13.1–65.5%), and estimates were similar after excluding the high-attrition cohort (35.0%) or the cohort with derived death counts (37.5%). The broader analysis adding clinically restricted, ICU-restricted, and non-standard-definition cohorts (16 cohorts) yielded 36.4% (95% CI 28.1–45.6; prediction interval 12.1–70.4%) (Table 2).

Eleven direct cohorts contributed to the primary exact 90-day analysis (3167 participants; 1380 deaths). Study-specific estimates ranged from 28.8% to 75.9%, and pooled mortality was 50.2% (95% CI 39.5–60.8), with I^2^ = 95.5%, τ^2^ = 0.382, and a prediction interval of 19.2–81.0% (Figure 2B). Excluding the high-attrition cohort (47.2%) or cohorts with derived death counts (47.9%) had little effect, and the broader 90-day/three-month analysis (19 cohorts) yielded 51.9% (95% CI 42.6–61.0; prediction interval 18.8–83.4%). In the post hoc framework-stratified analysis, APASL/AARC-defined cohorts contributed six studies to each primary analysis and EASL-CLIF-defined cohorts five at 28/30 days and three at 90 days; the cohort of Xi et al. [62] was split into its APASL-only and EASL-CLIF subgroups. Pooled mortality was 34.2% (95% CI 22.5–48.2) versus 40.5% (95% CI 24.2–59.2) at 28/30 days and 48.4% (95% CI 29.9–67.2) versus 57.9% (95% CI 22.2–86.9) at 90 days for APASL/AARC- and EASL-CLIF-defined cohorts, respectively. The difference between frameworks was not statistically significant (*p* = 0.46 and *p* = 0.48), and substantial heterogeneity persisted within both strata (I^2^ 80.9–97.5%), indicating that the diagnostic framework alone did not explain the between-study variation (Table 2; Supplementary Figure S6).

In the ICU-only cohort, crude in-hospital mortality was 45.2% (85/188) and differed markedly by precipitant: 60.2% with sepsis-associated ACLF versus 20.0% with nonseptic precipitants [23]. This estimate was not combined with fixed-time mortality. In the two additional ICU-restricted cohorts, 30-day mortality was 34.8% in alcohol-related cirrhosis with ACLF [47] and 74.7% (cumulative incidence) in severe alcohol-associated hepatitis–precipitated ACLF [55]; both entered only the broader sensitivity analyses.

**Table 2:** Primary and sensitivity analyses of short-term mortality.

Analysis	k	Patients/Deaths	Mortality (95% CI)	Heterogeneity	95% Prediction Interval
Primary analyses (direct, non-ICU-restricted cohorts, standard ACLF definitions)					
Primary 28/30-day	11	2497/855	36.0% (28.9–43.8)	I^2^ 87.6%; τ^2^ 0.177	17.3–60.3%
Primary exact 90-day	11	3167/1380	50.2% (39.5–60.8)	I^2^ 95.5%; τ^2^ 0.382	19.2–81.0%
Sensitivity analyses					
Strict 28-day (excluding 30-day endpoints)	8	1088/378	34.9% (25.1–46.1)	I^2^ 87.2%; τ^2^ 0.247	13.1–65.5%
28/30-day, excluding high-attrition cohort (Ray)	10	2364/794	35.0% (27.5–43.3)	I^2^ 87.7%; τ^2^ 0.179	16.3–59.8%
28/30-day, excluding derived counts (Broekhoven)	10	2424/840	37.5% (30.4–45.1)	I^2^ 86.2%; τ^2^ 0.147	19.2–60.1%
Broader 28/30-day ^a^	16	3009/1053	36.4% (28.1–45.6)	I^2^ 93.5%; τ^2^ 0.415	12.1–70.4%
90-day, excluding high-attrition cohort (Ray)	10	3034/1279	47.2% (37.7–57.0)	I^2^ 94.1%; τ^2^ 0.273	20.5–75.7%
90-day, excluding derived counts (Broekhoven, Ray)	9	2961/1249	47.9% (37.2–58.8)	I^2^ 95.0%; τ^2^ 0.301	19.4–77.8%
Broader 90-day/three-month ^b^	19	3518/1588	51.9% (42.6–61.0)	I^2^ 94.7%; τ^2^ 0.503	18.8–83.4%
ACLF framework-stratified analyses of the primary sets (post hoc) ^c^					
28/30-day: APASL/AARC	6	1826/626	34.2% (22.5–48.2)	I^2^ 91.7%; τ^2^ 0.260	11.0–68.6%
28/30-day: EASL-CLIF	5	375/149	40.5% (24.2–59.2)	I^2^ 80.9%; τ^2^ 0.283	11.5–78.2%
28-day: WGO	1	296/80	27.0% (raw proportion)	Not applicable	Not calculated
90-day: APASL/AARC	6	2511/1051	48.4% (29.9–67.2)	I^2^ 97.5%; τ^2^ 0.530	11.0–87.7%
90-day: EASL-CLIF	3	277/170	57.9% (22.2–86.9)	I^2^ 86.0%; τ^2^ 0.356	6.3–96.5%
90-day: WGO/Chinese criteria	1/1	296/116; 83/43	39.2%; 51.8% (raw)	Not applicable	Not calculated
Descriptive					
ICU in-hospital mortality (von Maldeghem et al.)	1	188/85	45.2% (raw proportion)	Not applicable	Not calculated

All pooled estimates from REML random-effects models on the logit scale with Hartung–Knapp confidence intervals. Primary 28/30-day set: Broekhoven, Castaño-Jiménez, Duseja, Giri, Lee, Matovic Zaric, Ray, Saini, Tong, Xi, Zhang. Primary 90-day set: Broekhoven, Castaño-Jiménez, Duseja, Giri, Jindal, Ray, Saini, Sonika, Tong, Yao, Zhang. ^a^ Adds Peng (infection-restricted), Slyvka (source-defined ACLF), Igna and Patidar (ICU), and Murakami (Japanese criteria including probable cases). ^b^ Adds Kulkarni (pilot), Mehta, Katoonizadeh, Chen, and Pati (three-month endpoints), Patidar (ICU), and Murakami and Kamezaki (Japanese criteria including extended/probable cases). ^c^ Xi et al. was split into its APASL-only (17/90) and EASL-CLIF (31/64) subgroups; test for subgroup difference (APASL/AARC vs. EASL-CLIF; mixed-effects meta-regression with Hartung–Knapp adjustment): *p* = 0.46 at 28/30 days and *p* = 0.48 at 90 days; these analyses were added post hoc in response to peer review. Derived counts: Broekhoven (Kaplan–Meier), Ray 90-day (subtraction), Patidar (cumulative incidence), Murakami and Kamezaki (Kaplan–Meier), Katoonizadeh (transplant-free survival). ICU in-hospital mortality was not pooled with fixed-time endpoints. ACLF, acute-on-chronic liver failure; AARC, APASL ACLF Research Consortium; APASL, Asian Pacific Association for the Study of the Liver; CI, confidence interval; EASL-CLIF, European Association for the Study of the Liver–Chronic Liver Failure Consortium; ICU, intensive care unit; k, number of cohorts; REML, restricted maximum likelihood; WGO, World Gastroenterology Organisation.

### 3.3. Clinical and Organ-Failure Predictors

#### 3.3.1. ACLF Severity, Organ Failure, and Renal Dysfunction

Predictor findings in Section 3.3, Section 3.4 and Section 3.5 derive from a structured qualitative synthesis; no pooled predictor effects were estimated. Estimates from supportive populations are explicitly identified as such and are used only to corroborate or contextualize findings from direct alcohol-related ACLF cohorts (Table 3 and Table 4).

**Figure 3:**
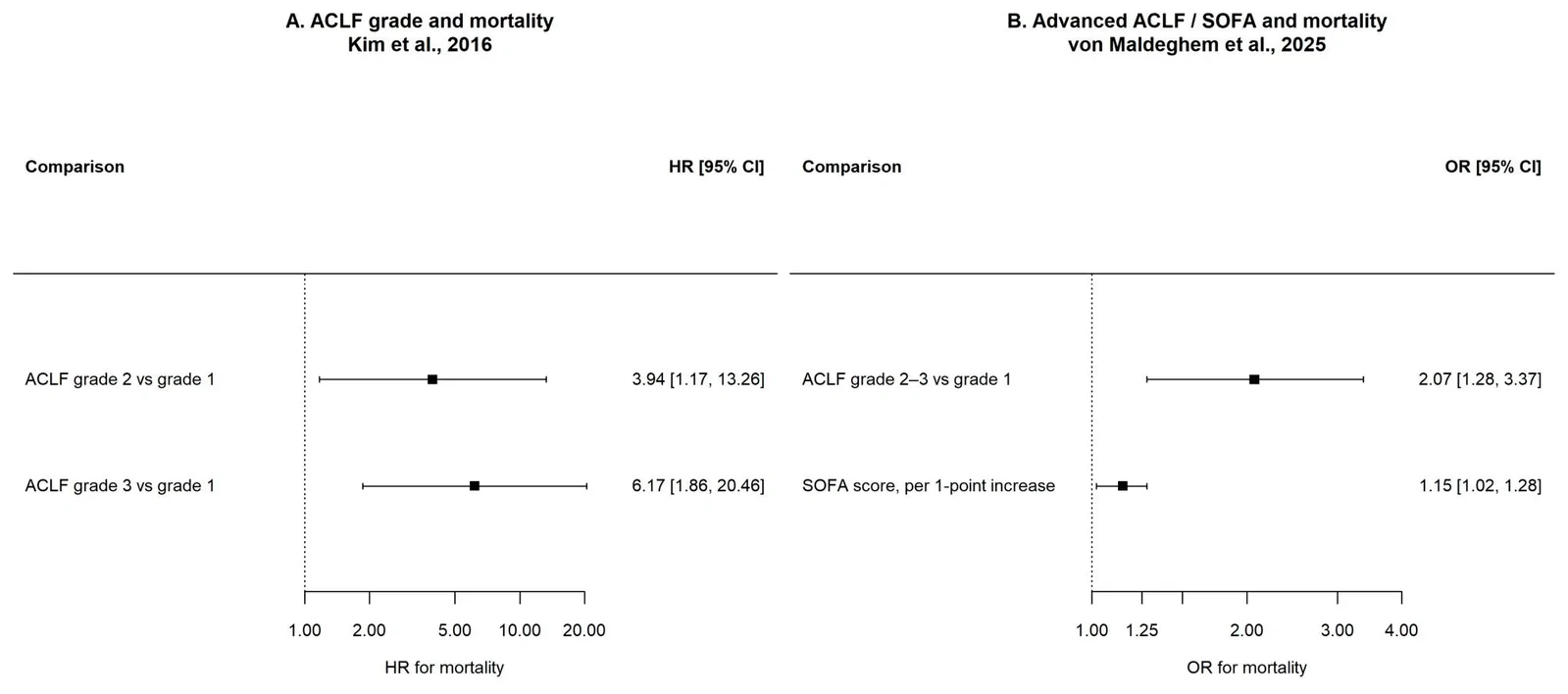
Association of ACLF severity and organ-failure burden with mortality. Panel (**A**) shows hazard ratios for ACLF grades 2 and 3 versus grade 1. Panel (**B**) shows separate adjusted odds-ratio models for advanced ACLF and SOFA score in an ICU cohort. HRs and ORs were displayed separately and were not pooled. Both panels represent direct alcohol-related ACLF evidence (Kim et al. [13]; von Maldeghem et al. [23]).

Greater ACLF severity was one of the most consistent prognostic signals. In the direct alcohol-related ACLF cohort of Kim et al. [13], grade 2 versus grade 1 was associated with HR 3.94 (95% CI 1.17–13.26), and grade 3 with HR 6.17 (95% CI 1.86–20.46). In the ICU cohort, grades 2–3 versus grade 1 were associated with adjusted OR 2.07 (95% CI 1.28–3.37), while each SOFA point increased the odds of in-hospital death by 15% in a separate model [23] (Figure 3). Sersté et al. [19] reported a parallel 28-day cumulative mortality gradient of 30.8%, 58.3%, and 72.4% across prevalent grades 1, 2, and 3 (Supplementary Figure S2). In a mixed-etiology long-term cohort, adjusted time ratios also shortened progressively with ACLF grade (Supplementary Figure S4), but these estimates were not combined with fixed-time mortality effects. Additional direct cohorts showed the same gradient: four-week mortality was 14.6%, 59.1%, and 83.6% for ACLF grades 1–3 among patients managed without transplantation [51]; 30-day mortality was 7.1%, 35.3%, and 80.0% across grades 1–3 in an ICU cohort [47]; and, within severe alcohol-associated hepatitis–precipitated ACLF in the ICU, grade 3 versus grade 1 was associated with sHR 2.27 (95% CI 1.20–4.31) [55] (Table 3).

Renal dysfunction was frequent and, although creatinine and kidney failure are embedded in MELD, AARC, CLIF-C, and SOFA, two direct cohorts provided adjusted renal-specific estimates: each 1 mg/dL increase in serum creatinine was associated with HR 1.14 (95% CI 1.02–1.27) for in-hospital death in alcohol-related ACLF [60], and creatinine was the only independent predictor of 90-day mortality in the ACLF subgroup of a first-decompensation cohort (OR 2.69, 95% CI 1.26–5.76) [46]. An overlapping registry report also identified creatinine as independently associated with 90-day mortality [50]. Because endpoints and effect measures differed, these estimates were not pooled.

#### 3.3.2. Hepatic Encephalopathy, Infection, and Shock

Hepatic encephalopathy showed a consistent adverse direction across adjusted analyses. Zhang et al. [24] reported HR 2.25 (95% CI 1.52–3.31) for 90-day mortality in the alcohol-related subgroup. Jindal et al. [11] reported an adjusted estimate of 1.63 (95% CI 1.36–2.25), although the publication inconsistently labeled the effect measure; it was therefore described neutrally and was not pooled. In mixed acute decompensation/ACLF cohorts, each increase in encephalopathy grade was associated with OR 1.69 (95% CI 1.20–2.38) [12], and the presence of encephalopathy was associated with OR 3.63 (95% CI 1.07–12.34) [16]. Glisic et al. [26] provided supportive ICU evidence but was not counted as an independent cohort because participant-level overlap with an included Serbian cohort could not be excluded. The two 90-day estimates are displayed in Supplementary Figure S3. In an additional direct alcohol-related ACLF cohort, early and advanced hepatic encephalopathy were independently associated with in-hospital death (HR 2.08, 95% CI 1.17–3.68, and HR 2.33, 95% CI 1.27–4.28) [60].

**Figure 4:**
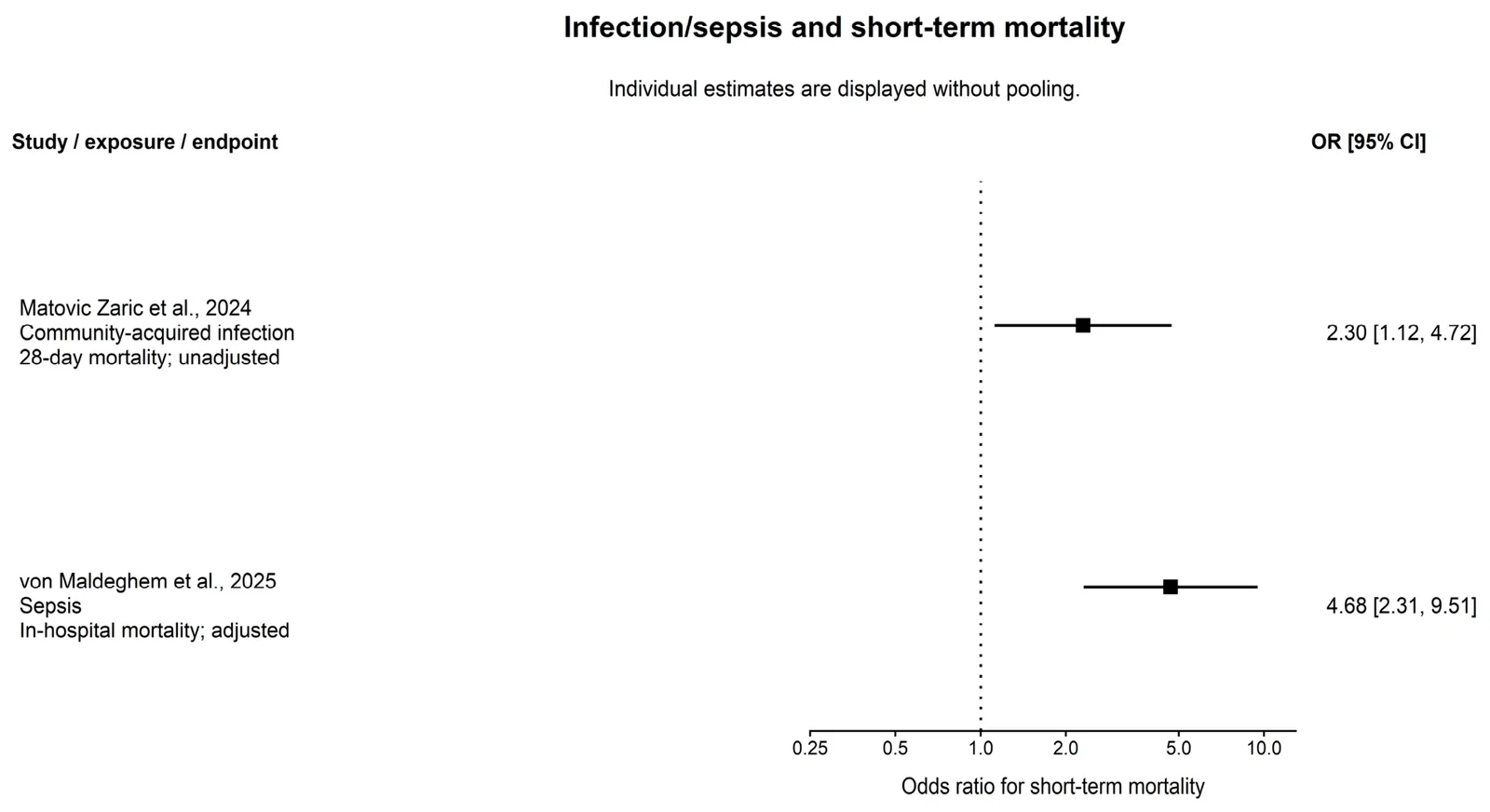
Individual odds-ratio estimates for infection/sepsis and short-term mortality. Community-acquired infection was evaluated in a combined acute decompensation/ACLF cohort and was unadjusted (Matovic Zaric et al. [16]; supportive/indirect evidence); sepsis was evaluated in an ICU alcohol-related ACLF cohort and was multivariable-adjusted (von Maldeghem et al. [23]; direct evidence). No pooled estimate was calculated because exposure definitions, populations, adjustments, and mortality endpoints differed.

Infection and sepsis were adverse across clinically distinct definitions. Baseline infection predicted poorer survival in the Korean alcohol-related ACLF cohort (HR 1.87, 95% CI 1.12–3.10) [13], and baseline bacterial infection predicted 90-day mortality in the alcohol-specific WGO subgroup (HR 1.83, 95% CI 1.02–3.30) [24]. In the ICU cohort, sepsis was associated with adjusted OR 4.68 (95% CI 2.31–9.51) for in-hospital death [23]. Infection developing during follow-up in severe alcohol-associated hepatitis produced a much larger time-varying association (adjusted HR 27.79, 95% CI 10.95–70.47) [19]. Because exposure timing, severity, endpoints, and populations differed, no common infection effect was calculated; OR estimates are shown in Figure 4 and HR estimates in Supplementary Figure S1. Infection at admission was also independently associated with in-hospital death in alcohol-related ACLF (HR 1.87, 95% CI 1.16–3.03) [60], and spontaneous bacterial peritonitis was associated with 30-day mortality in an ICU cohort [47].

Shock independently predicted 28-day mortality in infection-associated ALD-ACLF (OR 6.33, 95% CI 1.53–26.10) [18]. Other markers of circulatory and portal-hemodynamic dysfunction were also informative: a hepatic venous pressure gradient of at least 20 mmHg was associated with an adjusted effect of 1.86 (95% CI 1.31–2.37) in severe alcohol-associated hepatitis [11], while norepinephrine concentration, intrahepatic vascular resistance, and hepatic venous pressure gradient showed AUROCs of 0.91, 0.85, and 0.80, respectively, in a small exploratory cohort [17].

### 3.4. Biochemical, Inflammatory, and Novel Biomarker Predictors

Bilirubin, INR, creatinine, sodium, and albumin commonly tracked disease severity, but most lost independent significance when organ-failure variables or composite scores were included. Albumin was independently protective in one large alcohol-specific subgroup (HR 0.95 per g/L, 95% CI 0.90–1.00) [24]. In a 25-patient pilot study, total protein was associated with lower 90-day mortality (HR 0.03, 95% CI 0.003–0.36), but the estimate was imprecise and vulnerable to overfitting [15]. In a direct ALD-ACLF cohort, white blood cell count, total bilirubin, and prothrombin time dichotomized at data-derived cutoffs were independently associated with 90-day mortality (OR 4.99, 8.03, and 16.30, respectively) [63].

Ammonia was independently associated with 28-day death in infected ALD-ACLF (OR 1.027 per unit, 95% CI 1.006–1.049), and a model combining ammonia, MELD, and shock achieved AUROC 0.861 versus 0.776 for MELD alone [18]. Higher neutrophil percentage predicted 28-day mortality in the alcohol-related WGO subgroup (HR 1.05 per percentage point, 95% CI 1.01–1.08) [24]. Log-transformed leukocyte count predicted 30-day mortality in a combined acute decompensation/ACLF model (OR 3.81, 95% CI 1.03–14.03) [12]. The updated prospective cohort additionally identified systemic inflammatory response syndrome, serum lactate, and recent alcohol intake as candidate predictors of 15-day mortality; however, the data-derived thresholds, effect coding, and lack of external validation limit their immediate clinical application [25].

Novel biomarkers were promising but not ready for routine use. GP73 was independently associated with both 28- and 90-day mortality in 100 ALD-ACLF patients; the highest versus lowest tertile yielded HR 3.52 (95% CI 1.15–10.79) at 90 days, and GP73 AUROCs were 0.739 and 0.711 [21]. In a 25-patient cohort, A-FABP was associated with higher mortality (HR 1.27 per ng/mL), whereas L-FABP was inversely associated (HR 0.69) [15]. Decorin remained protective in a combined acute decompensation/ACLF model (OR 0.10, 95% CI 0.02–0.56), but the model was not ACLF-specific [12]. Circulating monocyte phenotypes were linked to a composite poor outcome in only six events and were considered mechanistic rather than validated predictors [22]. Further candidates included serum interleukin-6 (>23.3 pg/mL; HR 5.70, 95% CI 2.24–14.5, for 6-month mortality) [53], presepsin and procalcitonin in an ICU cohort [47], the CT-derived liver-to-abdominal area ratio (HR 1.067 per unit for three-month mortality) [44], and intestinal microbiota dominance indices [43]; all were derived in small single-center cohorts without external validation. In an overlapping registry report, diabetes mellitus was associated with 90-day mortality (HR 1.74, 95% CI 1.26–2.40) [50].

### 3.5. Prognostic Scores

**Figure 5:**
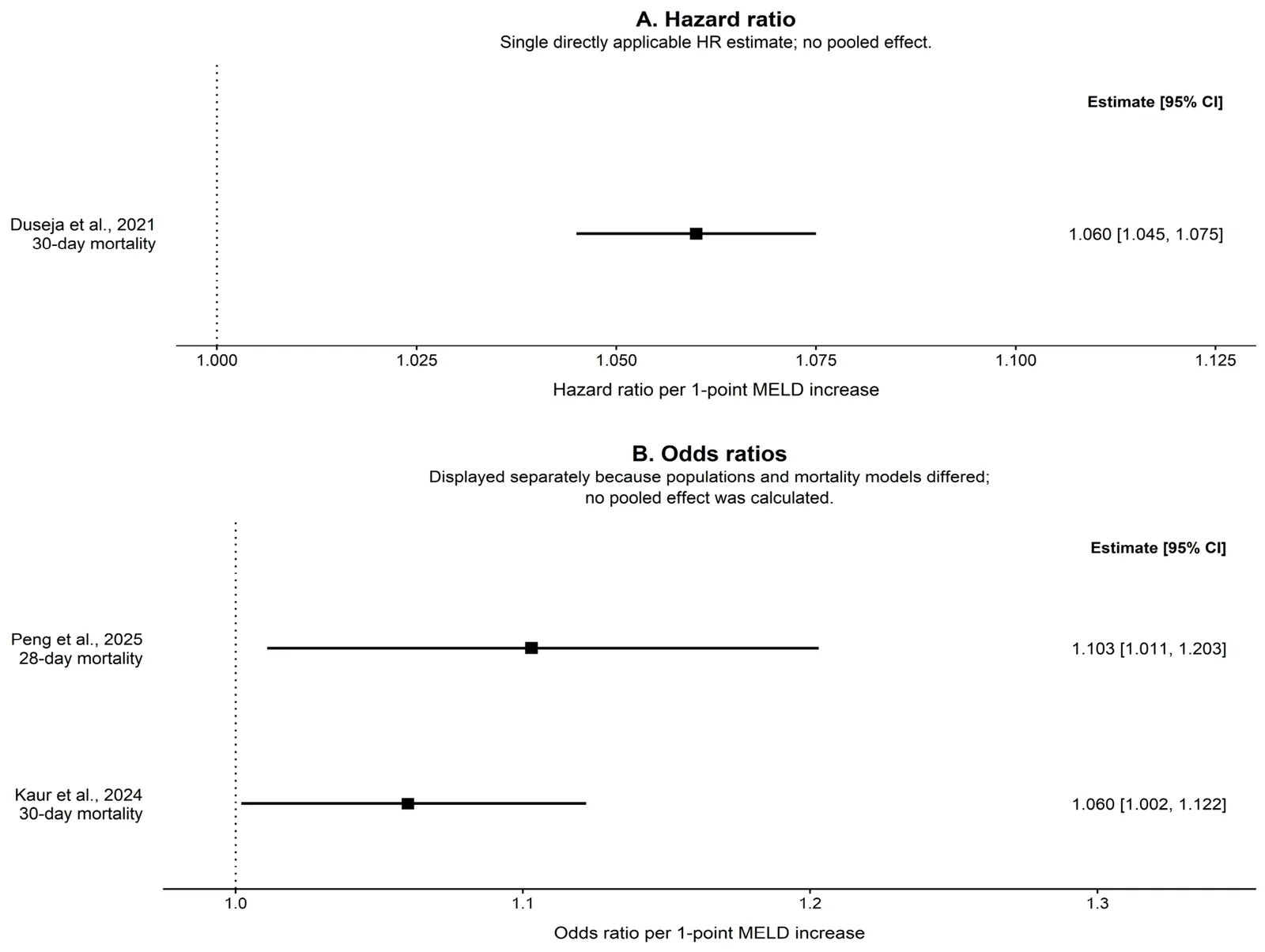
Association between MELD score and short-term mortality. Panel (**A**) shows the hazard ratio for 30-day mortality per one-point increase in MELD. Panel (**B**) shows odds ratios for 28/30-day mortality per point. HRs and ORs were displayed separately and were not pooled. Panel (**A**) (Duseja et al. [9]) and the infected ALD-ACLF cohort in Panel (**B**) (Peng et al. [18]) represent direct alcohol-related ACLF evidence, whereas the combined acute decompensation/ACLF cohort in Panel (**B**) (Kaur et al. [12]) represents supportive/indirect evidence.

MELD showed a consistent adverse direction. In the large AARC registry, each one-point increase was associated with HR 1.06 (95% CI 1.05–1.08) for 30-day mortality [9]. In the infected ALD-ACLF cohort, the corresponding OR was 1.103 (95% CI 1.011–1.203) for 28-day death [18], and in a combined acute decompensation/ACLF cohort it was OR 1.06 (95% CI 1.00–1.12) [12] (Figure 5). The two OR estimates showed a consistent adverse direction but were displayed separately and were not pooled because the populations and mortality models differed.

**Figure 6:**
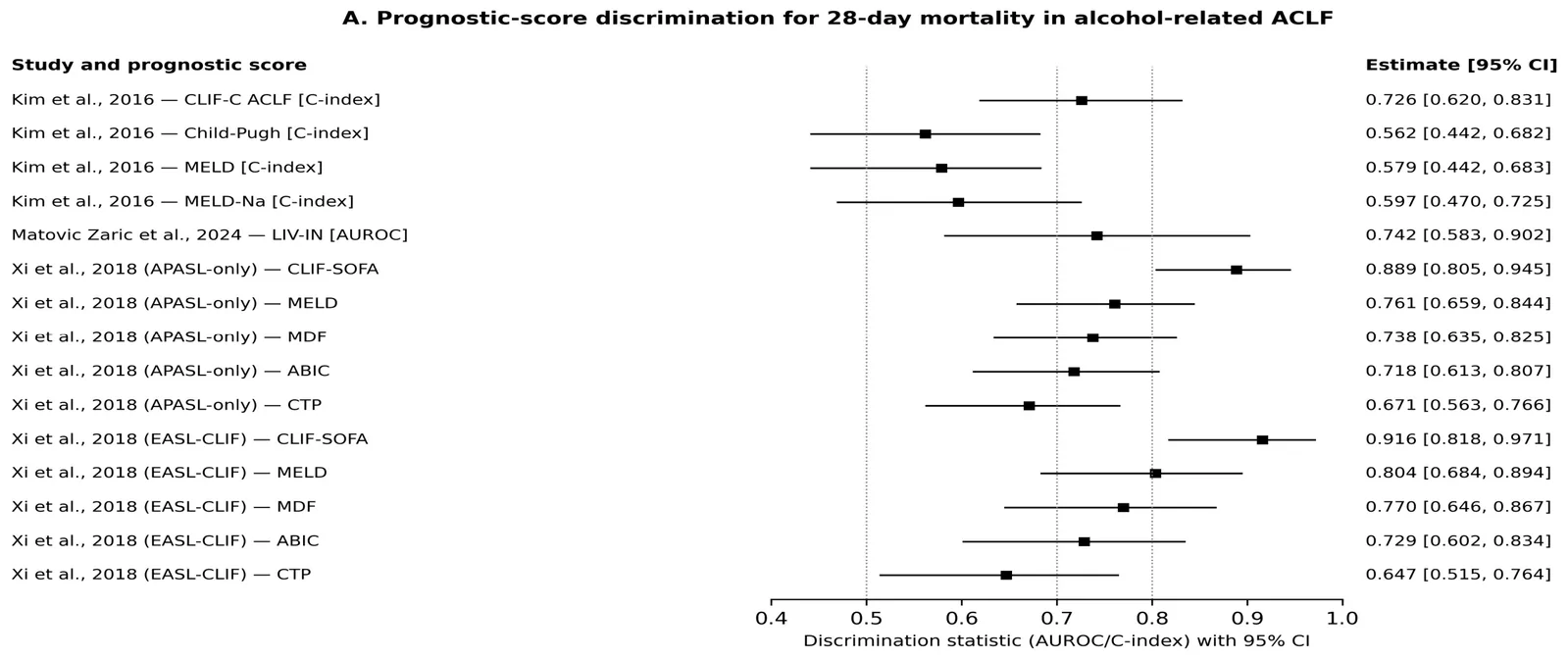

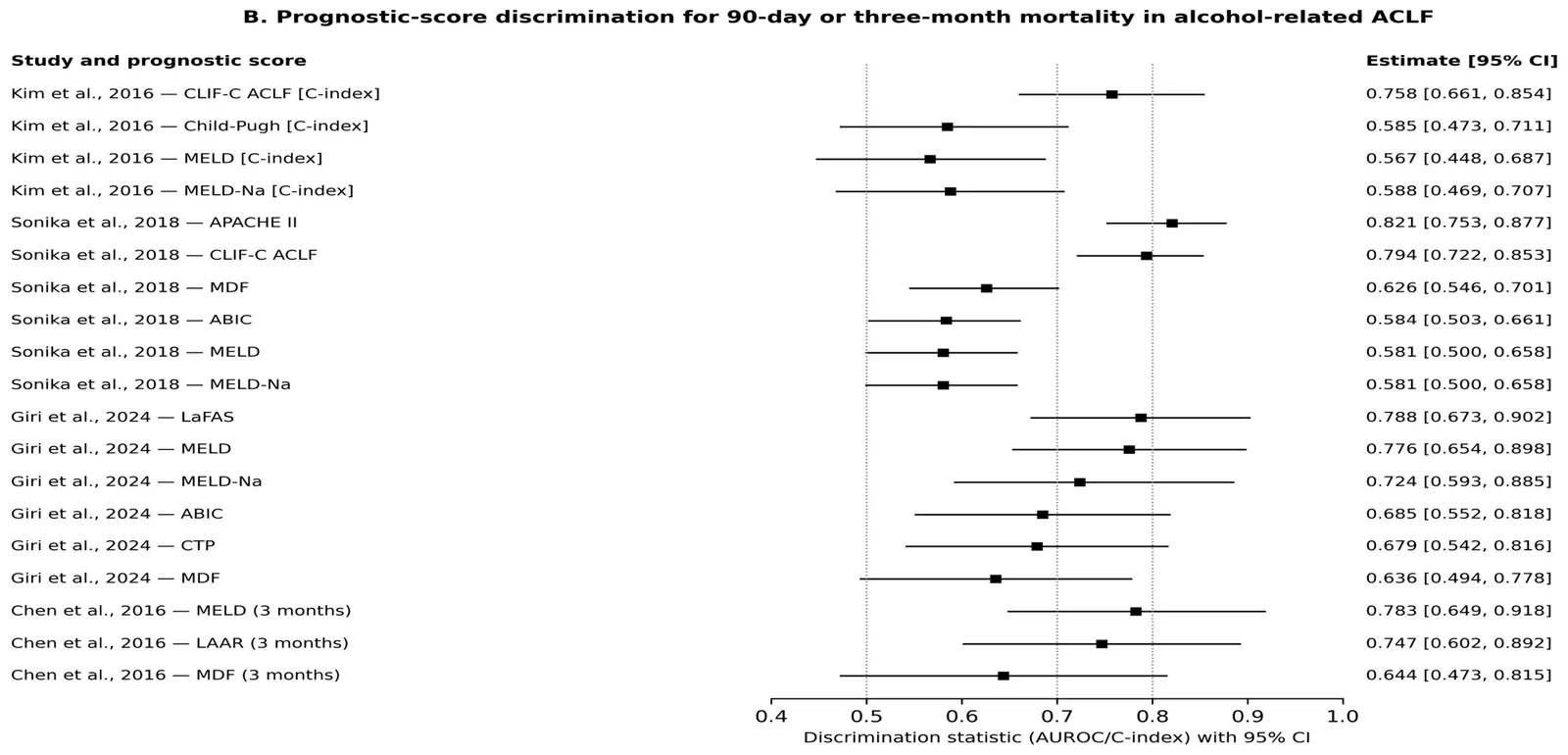
Discrimination of prognostic scores for short-term mortality in direct alcohol-related ACLF cohorts. (**A**) Twenty-eight-day mortality [13,16,62]. (**B**) Ninety-day or three-month mortality [13,44,46,60]. C-index and AUROC estimates are shown on a common descriptive scale but were not pooled; estimates from different cohorts are not head-to-head comparisons. Supportive estimates from populations without a separately analyzed ACLF subgroup are shown in Supplementary Figure S5. AARC, APASL ACLF Research Consortium; ABIC, age, bilirubin, INR, and creatinine score; ACLF, acute-on-chronic liver failure; APACHE II, Acute Physiology and Chronic Health Evaluation II; APASL, Asian Pacific Association for the Study of the Liver; AUROC, area under the receiver operating characteristic curve; CI, confidence interval; C-index, concordance index; CLIF-C ACLF, Chronic Liver Failure Consortium acute-on-chronic liver failure score; CLIF-SOFA, Chronic Liver Failure–Sequential Organ Failure Assessment; CTP, Child–Turcotte–Pugh score; EASL-CLIF, European Association for the Study of the Liver–Chronic Liver Failure Consortium; INR, international normalized ratio; LAAR, liver-to-abdominal area ratio; LaFAS, lactate-free AARC ACLF score; LIV-IN, Liver Mortality Inpatients prognostic score; MDF, Maddrey discriminant function; MELD, Model for End-Stage Liver Disease; MELD-Na, MELD–sodium.

CLIF-C ACLF provided the most consistent discrimination in EASL-CLIF-defined alcohol-related ACLF. Kim et al. [13] reported C-indices of 0.726 at 28 days and 0.758 at 90 days, exceeding Child–Pugh, MELD, and MELD-Na. Zhang et al. [24] found HR 1.07 (95% CI 1.04–1.11) per CLIF-C ACLF point for 28-day mortality; in the WGO type C subgroup, its AUROCs were 0.758 and 0.734 at 28 and 90 days. Figure 6 compares discrimination estimates in direct alcohol-related ACLF cohorts.

In additional direct cohorts, APACHE II and CLIF-C ACLF provided the highest 90-day AUROCs (0.821 and 0.794) in EASL-CLIF-defined alcohol-related ACLF [60]; CLIF-SOFA outperformed MELD, MDF, ABIC, and Child–Pugh for 28-day mortality in both APASL-only and EASL-CLIF subgroups (AUROC 0.889 and 0.916) [62]; and the lactate-free AARC score and MELD performed best in an APASL-defined subgroup (90-day AUROC 0.788 and 0.776) [46].

AARC was independently associated with mortality in two APASL-defined cohorts: HR 1.09 per point (95% CI 1.06–1.12) in the 1216-patient registry [9] and HR 3.30 (95% CI 1.15–9.54) in the small pilot cohort [15]. Child–Pugh generally performed less well. The study-derived LIV-IN score achieved AUROC 0.742 in a 38-patient ACLF subgroup but lacked external validation [16]. In a supportive severe alcohol-associated hepatitis cohort without a separate ACLF denominator, CLIF-C OF had the highest 28-day AUROC (0.89), whereas MELD-Na had the highest 90-day AUROC (0.87) (Supplementary Figure S5).

**Table 3:** Representative adjusted prognostic associations, with evidence type (direct vs. supportive/indirect) indicated for each estimate.

Predictor/Domain	Study	Evidence Type	Endpoint	Adjusted Association (95% CI)	Interpretive Limitation
ACLF grade	Kim et al. [13]	Direct	Overall survival	Grade 2 vs. 1: HR 3.94 (1.17–13.26); grade 3 vs. 1: HR 6.17 (1.86–20.46)	Direct, graded association; wide CIs
Advanced ACLF/SOFA	von Maldeghem et al. [23]	Direct	In-hospital	Grades 2–3 vs. 1: OR 2.07 (1.28–3.37); SOFA per point: OR 1.15 (1.02–1.28)	Separate ICU models
Baseline infection	Kim et al. [13]; Zhang et al. [24]	Direct	Survival/90 days	HR 1.87 (1.12–3.10); HR 1.83 (1.02–3.30)	Direct alcohol-related ACLF
Sepsis	von Maldeghem et al. [23]	Direct	In-hospital	OR 4.68 (2.31–9.51)	ICU-specific, strong association
Time-varying infection	Sersté et al. [19]	Supportive (severe AH)	28 days	aHR 27.79 (10.95–70.47)	Supportive severe-AH cohort; not baseline exposure
Hepatic encephalopathy	Zhang et al. [24]; Jindal et al. [11]	Direct [24]; near-direct [11]	90 days	HR 2.25 (1.52–3.31); adjusted estimate 1.63 (1.36–2.25)	Jindal source is internally inconsistent for effect label (HR/OR), upper CI (2.25/2.35), and HE timing; Table 3 CI was used
Shock	Peng et al. [18]	Direct	28 days	OR 6.33 (1.53–26.10)	Infection-restricted cohort; imprecise
MELD per point	Duseja et al. [9]; Peng et al. [18]; Kaur et al. [12]	Direct [9,18]; supportive [12]	28/30 days	HR 1.06 (1.05–1.08); OR 1.103 (1.011–1.203); OR 1.06 (1.00–1.12)	Consistent direction; mixed populations/effect measures
AARC score	Duseja et al. [9]; Kulkarni et al. [15]	Direct	30/90 days	HR 1.09 per point (1.06–1.12); HR 3.30 (1.15–9.54)	Framework-specific; pilot estimate imprecise
CLIF-C ACLF per point	Zhang et al. [24]	Direct	28 days	HR 1.07 (1.04–1.11)	Direct alcohol-specific subgroup
Neutrophil percentage	Zhang et al. [24]	Direct	28 days	HR 1.05 per percentage point (1.01–1.08)	Inflammatory signal beyond CLIF-C ACLF
Albumin	Zhang et al. [24]	Direct	90 days	HR 0.95 per g/L (0.90–1.00)	Modest protective association
Ammonia	Peng et al. [18]	Direct	28 days	OR 1.027 per unit (1.006–1.049)	Infection-associated ALD-ACLF
GP73	Tong et al. [21]	Direct	28/90 days	HR 1.01 per ng/mL; highest vs. lowest tertile HR 3.52 (1.15–10.79) at 90 days	Single-center; no external validation
SIRS	Saini et al. [25]	Direct	15 days	Present vs. absent: HR 9.5	Single-center, data-derived model; no external validation
ACLF grade (severe AH-precipitated ACLF)	Patidar et al. [55]	Direct (ICU)	180 days	Grade 2 vs. 1: sHR 1.15 (0.54–2.49); grade 3 vs. 1: sHR 2.27 (1.20–4.31)	Unadjusted competing-risk; ICU-restricted
Hepatic encephalopathy	Sonika et al. [60]	Direct	In-hospital	Early HE: HR 2.08 (1.17–3.68); advanced HE: HR 2.33 (1.27–4.28)	In-hospital endpoint
Infection at admission	Sonika et al. [60]	Direct	In-hospital	HR 1.87 (1.16–3.03)	In-hospital endpoint
Serum creatinine	Sonika et al. [60]; Giri et al. [46]	Direct	In-hospital; 90 days	HR 1.14 per mg/dL (1.02–1.27); OR 2.69 (1.26–5.76)	Different endpoints and effect measures
WBC, bilirubin, prothrombin time	Yao et al. [63]	Direct	90 days	OR 4.99 (1.31–19.05); OR 8.03 (2.16–29.85); OR 16.30 (4.08–65.16)	Data-derived cutoffs; wide CIs
LAAR; MELD	Chen et al. [44]	Direct	3 months	HR 1.067 per unit (1.025–1.111); HR 1.103 per point (1.016–1.197)	Small cohort; reported as OR
IL-6 > 23.3 pg/mL; day-4 MELD > 25	Murakami et al. [53]	Direct (non-standard definition)	6 months	HR 5.70 (2.24–14.5); HR 2.72 (1.05–7.09)	Japanese criteria incl. probable cases
Presepsin; procalcitonin	Igna et al. [47]	Direct (ICU)	30 days	OR 4.01 (3.13–6.46); OR 3.67 (2.31–5.81)	Adjustment and units unclear
Diabetes mellitus	Kumar et al. [50]	Overlapping registry report	90 days	HR 1.74 (1.26–2.40)	Contextual only; overlaps with [9]

Effect measures are reproduced as reported and are not directly interchangeable. Evidence type: direct, study restricted to alcohol-related ACLF or providing an extractable alcohol-related ACLF subgroup; near-direct, severe alcohol-associated hepatitis cohort in which most participants had ACLF; supportive/indirect, severe alcohol-associated hepatitis, mixed-etiology, or combined acute decompensation/ACLF population without a separable alcohol-related ACLF analysis. AARC, APASL ACLF Research Consortium; ACLF, acute-on-chronic liver failure; aHR, adjusted hazard ratio; AH, alcohol-associated hepatitis; ALD, alcohol-related liver disease; APASL, Asian Pacific Association for the Study of the Liver; CI, confidence interval; CLIF-C ACLF, Chronic Liver Failure Consortium acute-on-chronic liver failure score; GP73, Golgi protein 73; HE, hepatic encephalopathy; HR, hazard ratio; ICU, intensive care unit; IL-6, interleukin-6; incl., including; LAAR, liver-to-abdominal area ratio; MELD, Model for End-Stage Liver Disease; OR, odds ratio; sHR, subdistribution hazard ratio; SIRS, systemic inflammatory response syndrome; SOFA, Sequential Organ Failure Assessment; vs, versus; WBC, white blood cell count.

### 3.6. Risk of Bias and Certainty of Evidence

**Figure 7:**
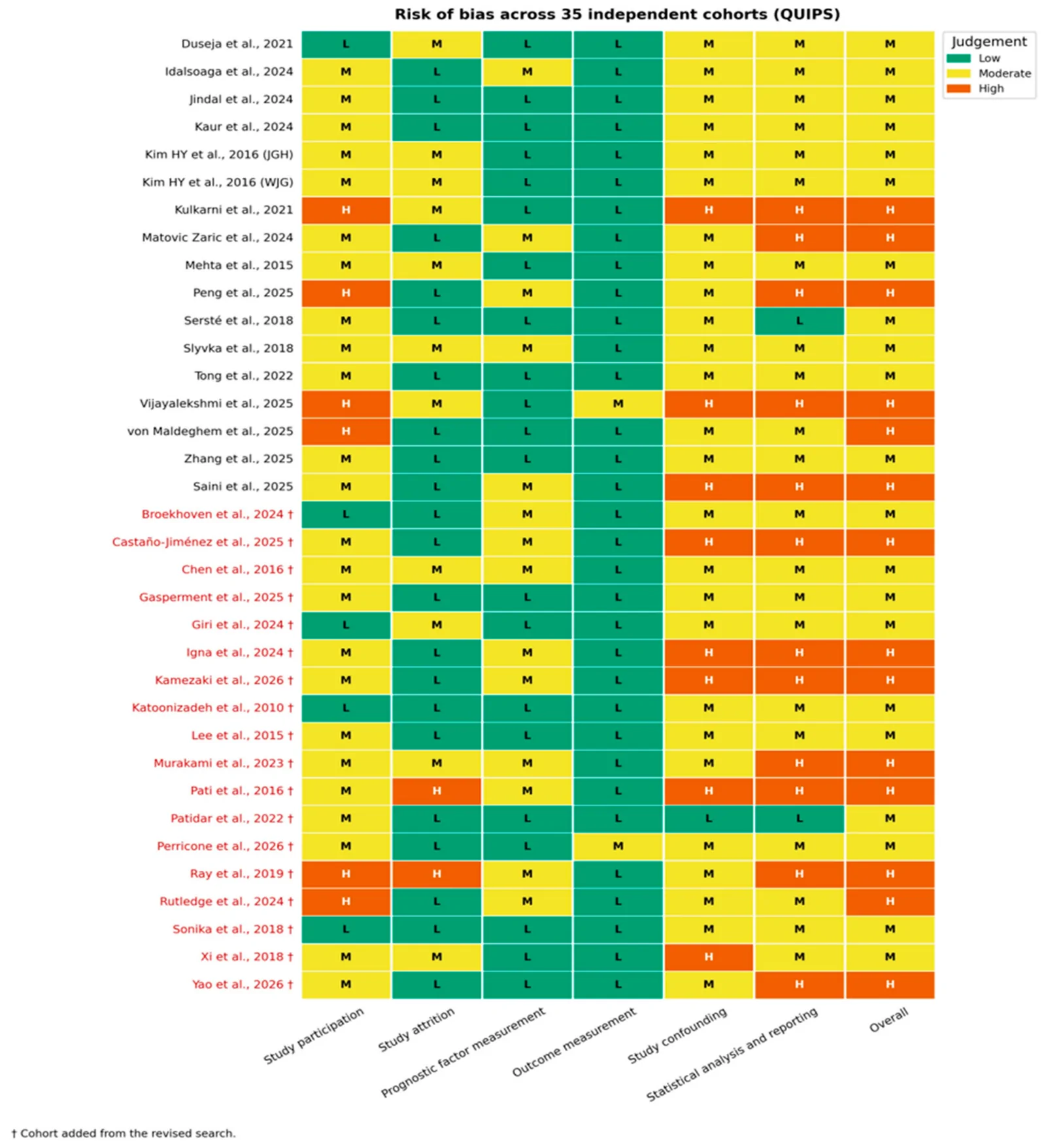
Risk of bias across 35 independent cohorts [9,10,11,12,13,14,15,16,17,18,19,20,21,22,23,24,25,42,43,44,45,46,47,48,49,51,53,54,55,56,57,58,60,62,63] using QUIPS [33]. Green, low risk; yellow, moderate risk; red, high risk. Cohorts added from the revised search are marked †. No study had low overall risk of bias.

No study had low overall risk of bias. Of the 35 independent cohorts, 21 (60.0%) were rated moderate and 14 (40.0%) high. Outcome measurement was low risk in 33 cohorts and prognostic-factor measurement in 21. The most frequent concerns were selected study participation, attrition, incomplete control of confounding, small event counts, data-driven modeling, overfitting, and limited internal or external validation. High overall risk was assigned to Kulkarni et al. [15], Matovic Zaric et al. [16], Peng et al. [18], Vijayalekshmi et al. [22], von Maldeghem et al. [23], Saini et al. [25], Castaño-Jiménez et al. [43], Igna et al. [47], Kamezaki et al. [48], Murakami et al. [53], Pati et al. [54], Ray et al. [57], Rutledge et al. [58], and Yao et al. [63] (Figure 7).

Certainty was moderate only for the single large, precise 30-day MELD HR estimate. Despite additional direct cohorts, evidence for infection/sepsis, hepatic encephalopathy, MELD expressed as an OR, ACLF severity/organ-failure burden, and renal dysfunction remained very low because of heterogeneous definitions, mixed effect measures and endpoints, small information size, and residual confounding (Table 4). Publication bias could not be assessed formally for any predictor domain because no domain contained at least 10 independent studies; funnel-plot methods were not applied to the mortality proportions because they are unreliable for proportion data.

**Table 4:** GRADE summary for major predictor domains, with the evidence type of contributing studies.

Domain	Evidence Base	Summary Effect	Heterogeneity	Certainty	Main Reason for Downgrading
Infection or sepsis	5 studies; 920 participants (direct [13,23,24,60]; supportive [19])	Not pooled; all effects adverse	Not estimable	Very low	Heterogeneous timing/severity, effect measures, populations, endpoints
Hepatic encephalopathy	5 studies; 1607 participants (direct [24,60]; near-direct [11]; supportive [12,16])	Not pooled; consistent adverse direction	Not estimable	Very low	Binary vs. ordinal exposure; mixed HR/OR and mortality windows
MELD per point (OR)	2 studies; 265 participants (direct [18]; supportive [12])	ORs 1.103 and 1.06; not pooled	Not estimable	Very low	Only two clinically different cohorts; one mixed AD/ACLF population; residual confounding
MELD per point (HR)	1 study; 1216 participants (direct [9])	HR 1.06 (95% CI 1.05–1.08)	One study	Moderate	Direct and precise, but observational and unreplicated
ACLF severity/organ failure	5 studies; 765 participants (direct [13,23,47,51,55])	Not pooled; graded adverse effects and consistent crude mortality gradients	Not estimable	Very low	Different contrasts, effect measures, settings, and shared reference groups
Renal dysfunction	2 studies; 231 participants (direct [46,60]); overlapping registry report [50]	Creatinine: HR 1.14 per mg/dL (in-hospital) and OR 2.69 (90 days); not pooled	Not estimable	Very low	Different endpoints and effect measures; overlap with composite scores; small information size
SIRS, lactate, and recent alcohol intake	1 study; 227 participants (direct [25])	Candidate predictors of 15-day mortality; no pooled effect	Not estimable	Very low	Single-center study; data-derived thresholds and effect coding; no external validation

Formal publication-bias assessment was not possible for any domain because fewer than 10 independent studies contributed. Evidence type is defined in the footnote to Table 3; supportive/indirect evidence contributed to downgrading for indirectness. ACLF, acute-on-chronic liver failure; AD, acute decompensation; CI, confidence interval; GRADE, Grading of Recommendations Assessment, Development and Evaluation; HR, hazard ratio; MELD, Model for End-Stage Liver Disease; OR, odds ratio; SIRS, systemic inflammatory response syndrome.

## 4. Discussion

### 4.1. Principal Findings

This review synthesized 35 independent cohorts of alcohol-related ACLF. Pooled mortality was 36.0% at 28/30 days and 50.2% at 90 days, but these averages conceal major variation: I^2^ was 87.6% and 95.5% in the primary models, and the prediction intervals extended from 17.3% to 60.3% and from 19.2% to 81.0%, respectively. These prediction intervals, rather than the pooled point estimates alone, are the most informative summary for clinical interpretation: they indicate that 28/30-day mortality in a new, comparable setting could plausibly range from approximately 1 in 6 to 3 in 5 patients, and 90-day mortality from approximately 1 in 5 to 4 in 5 patients. The pooled averages should therefore not be used as a fixed benchmark for an individual center, for audit targets, or for patient-level prognosis. This dispersion is consistent with the differences in clinical setting, case mix, and handling of transplantation and censoring discussed in Section 4.5; notably, it was not explained by the ACLF diagnostic framework. Comparable adjusted estimates were rarely available, so pooled effect sizes could not be generated for any individual prognostic factor; the predictor findings therefore derive from a structured qualitative synthesis based on the direction, consistency, adjustment, and directness of reported associations, not from quantitative meta-analysis. Within this synthesis, the most reproducible adverse signals, each observed in at least one direct alcohol-related ACLF cohort, were a greater number or grade of organ failures, infection or sepsis, hepatic encephalopathy, renal dysfunction, shock, and higher multicomponent severity scores. Isolated laboratory abnormalities were less consistently independent, and most novel biomarkers were supported by one small or selected cohort.

The findings support a syndrome-based rather than single-marker approach to prognosis. Organ-failure burden showed a dose–response pattern across direct alcohol-related cohorts, and SOFA retained prognostic value in critically ill patients. Renal dysfunction, although embedded within ACLF classification and several prognostic scores, retained independent associations with mortality in two direct cohorts [46,60]. The principal conclusions of this review rest on direct alcohol-related ACLF evidence. Both primary mortality meta-analyses were restricted to direct cohorts, and each of the most consistent adverse signals was observed in at least one direct cohort: organ-failure burden or ACLF grade [13,23,47,51,55], baseline infection or sepsis [13,23,24,60], hepatic encephalopathy [24,60] (with near-direct support [11]), renal dysfunction [46,60], shock [18], and MELD-, CLIF-C ACLF-, or AARC-based severity [9,13,15,18,24,46,60,62]. Supportive populations contributed corroborating or contextual findings only. Several observations rest solely on supportive evidence and should not be extrapolated to alcohol-related ACLF without confirmation: the magnitude of risk associated with infection developing during follow-up [19], leukocyte count and decorin [12], community-acquired infection and hepatic encephalopathy in combined acute decompensation/ACLF models [12,16], and the relative discrimination of CLIF-C OF and MELD-Na in severe alcohol-associated hepatitis [14]. The post hoc framework-stratified analysis showed no significant difference between APASL/AARC- and EASL-CLIF-defined cohorts at either time point, and marked heterogeneity persisted within both frameworks; cohorts defined by Japanese criteria, which include patients who do not meet EASL-CLIF criteria [48], were analyzed only in sensitivity analyses [48,53].

### 4.2. Infection, Neurological Dysfunction, and Dynamic Risk

Infection was consistently adverse, but effect magnitude depended on what was measured. Baseline bacterial infection produced moderate HRs, while sepsis in the ICU and infection emerging during follow-up produced much larger associations. This is biologically and clinically coherent: culture-negative infection, microbiologically confirmed infection, sepsis, and septic shock represent different exposures, and infection after ACLF onset can be both a cause and a consequence of worsening organ dysfunction. A single pooled infection effect would therefore be misleading.

Hepatic encephalopathy also remained adverse across direct and supportive analyses. It captures severe hepatic dysfunction, cerebral organ failure, systemic inflammation, and vulnerability to aspiration and infection, but it overlaps with ACLF grading. At the bedside, the key implication is not to choose between encephalopathy and grade, but to document neurological status explicitly and reassess it alongside renal, circulatory, and respiratory function.

A recently published, potentially overlapping ICU report from the same Serbian center evaluated 100 patients with alcohol-related ACLF and hepatic encephalopathy. Twenty-eight-day mortality was 55.7% among patients with West Haven grades I–II and 81.2% among those with grades III–IV, while higher-grade hepatic encephalopathy was associated with an increased hazard of death (HR 2.34, 95% CI 1.15–2.98) [26]. Because participant-level independence from the included Serbian cohort could not be established, this report was used only for contextual interpretation and was not treated as an independent unit in the quantitative synthesis.

Because ACLF changes rapidly, serial assessment is more defensible than relying on an isolated admission score. Deterioration during the first 24–72 h, a new infection, escalating vasopressor or ventilatory requirements, or increasing organ-failure count should trigger immediate risk reclassification. Conversely, early improvement may materially alter expected outcome and should prevent premature use of a baseline score as a futility threshold.

### 4.3. Prognostic Scores and Biomarkers

MELD had the most credible replicated direction of association, but its discrimination varied. CLIF-C ACLF was more consistent in EASL-CLIF-defined alcohol-related ACLF because it represents extrahepatic organ failure and inflammation rather than liver and renal laboratory values alone. AARC was prognostic in APASL-defined populations. These findings do not establish a universally superior score; they indicate that the score should match the diagnostic framework and intended time horizon. Additional direct cohorts reinforced the advantage of organ-failure-based scores (CLIF-C ACLF, CLIF-SOFA, APACHE II) over liver-specific scores (MDF, ABIC, Child–Pugh) [60,62], whereas the lactate-free AARC score and MELD performed well in an APASL-defined subgroup [46].

Novel biomarkers may eventually refine risk classification, but the evidence is not yet sufficient for routine use. GP73, ammonia-based models, fatty acid-binding proteins, decorin, and hemodynamic measures showed encouraging associations or discrimination. However, small samples, selected eligibility criteria, transformed predictor scales, stepwise modeling, incomplete calibration reporting, and lack of external validation create a high risk of optimism. Incremental AUROC gains alone are insufficient; future studies should evaluate calibration, reclassification, net benefit, and clinical impact. The same limitations apply to interleukin-6, presepsin, procalcitonin, the liver-to-abdominal area ratio, and microbiota-based indices identified in the updated search [43,44,47,53].

### 4.4. Clinical Implications

At admission, clinicians should document the ACLF definition and grade, calculate a framework-appropriate score, and assess infection or sepsis, hepatic encephalopathy, shock or vasopressor requirement, kidney injury, respiratory failure, and current organ-support needs. This integrated assessment is more reliable than any isolated biochemical marker. Infection surveillance should continue throughout hospitalization because incident infection can substantially change prognosis.

High-risk patients require early multidisciplinary involvement, including hepatology, critical care, infectious diseases, and transplant teams where appropriate. Prognostic estimates can guide monitoring intensity, ICU escalation, antimicrobial investigation, renal replacement or circulatory support, and timely transplant evaluation. They should not be used as stand-alone rules to deny treatment, particularly given the observational evidence base, heterogeneous definitions, and capacity for early clinical improvement.

### 4.5. Strengths and Limitations

Strengths include the explicit separation of direct alcohol-related ACLF evidence from supportive mixed or severe-hepatitis evidence, endpoint-specific mortality pooling, preservation of reported effect measures, use of REML random-effects models with Hartung–Knapp confidence intervals and prediction intervals, and independent QUIPS and GRADE assessment. The review also avoided converting ORs to HRs or pooling incompatible predictor units solely to increase the number of estimates.

The main limitation is clinical and methodological heterogeneity. I^2^ was 87.6% and 95.5% in the primary mortality models, and the 95% prediction intervals spanned approximately 17–60% and 19–81%, so the pooled estimates describe an average across heterogeneous settings rather than the expected mortality in any single setting. Framework-specific pooling was feasible for APASL/AARC- and EASL-CLIF-defined cohorts, but heterogeneity persisted within both strata, only single cohorts used WGO or Chinese national criteria, and cohorts using Japanese criteria were analyzed only in sensitivity analyses. Pooled effect estimates could not be generated for individual prognostic factors, so predictor conclusions rest on a structured qualitative synthesis and cannot be expressed as summary effect sizes. Studies used EASL-CLIF, APASL/AARC, WGO, Chinese, Japanese, or study-specific definitions; enrolled ward, ICU, infection-restricted, or severe-hepatitis populations; and handled transplantation and censoring inconsistently. All studies had moderate or high overall risk of bias, and several models had few events or no external validation. Death counts had to be derived from survival or cumulative-incidence estimates in six cohorts, and one cohort had high attrition; excluding these did not materially change the pooled estimates. Generalizability is limited by predominantly Asian and male cohorts. Patient-level data were unavailable for harmonized reanalysis, and publication bias could not be evaluated statistically. The targeted manual search identified two eligible reports. Saini et al. was treated as an independent cohort, whereas Glisic et al. was handled as a potentially overlapping secondary report because participant-level independence from the Serbian cohort could not be established; four further reports from the APASL ACLF Research Consortium registry or from the same center as an included cohort were handled in the same way. Identification of the two reports after the original database searches suggested that the original strategy had been overly restrictive; the search was therefore systematically revised and rerun in all three databases (Section 2.3), and it retrieved all previously included reports. Nevertheless, five potentially eligible reports could not be retrieved in full text, and eligible reports that are not indexed in these databases or not published in English may have been missed. The search procedures are reported in Section 2.3 and Supplementary Table S1 to allow their reproduction.

### 4.6. Research Priorities

Future studies should prospectively enroll clearly defined alcohol-related ACLF populations, state the diagnostic framework, use prespecified 28/30-day and 90-day endpoints, and report transplantation, terminal discharge, loss to follow-up, and competing events transparently. Predictor timing and units should be standardized, and serial models should capture improvement or progression during the first days. Prediction-model studies should use adequate event numbers, handle missing data and nonlinearity appropriately, and report discrimination, calibration, and decision-curve or clinical-utility measures. Development should be followed by temporal and geographic external validation and reporting consistent with TRIPOD and PROBAST [34,65]. Particular priorities are dynamic infection models, validation of biomarker panels against CLIF-C ACLF, MELD, and AARC, and impact studies testing whether model-guided triage or transplant referral improves patient-centered outcomes.

## 5. Conclusions

Alcohol-related ACLF carries substantial early mortality: across 11 direct cohorts for each endpoint, pooled mortality was 36.0% at 28/30 days and 50.2% at 90 days, although the wide 95% prediction intervals (17.3–60.3% and 19.2–81.0%, respectively) indicate that mortality in individual settings may differ substantially from these averages. Estimates did not differ significantly between APASL/AARC- and EASL-CLIF-defined cohorts. In a structured qualitative synthesis of direct alcohol-related ACLF evidence, the most consistent adverse signals were increasing organ-failure burden, infection or sepsis, hepatic encephalopathy, renal dysfunction, shock, and higher MELD-, CLIF-, or AARC-based severity. Pooled effect estimates could not be generated for individual predictors. Clinical risk stratification should therefore combine a framework-appropriate score with direct assessment of infection, neurological and circulatory dysfunction, renal and respiratory failure, and short-interval trajectory. Substantial heterogeneity, moderate or high risk of bias in all included cohorts, and predominantly very low-certainty predictor evidence preclude endorsement of a universal cutoff or a single model for every alcohol-related ACLF population. Prospective multicenter validation with standardized definitions and fixed mortality windows is required before newer biomarkers or models can be adopted routinely.

## Data Availability

No new primary data were generated in this study. This systematic review and meta-analysis extracted and quantitatively analyzed published aggregate data from the included studies. The study-level data used in the analyses (sample sizes, numbers of deaths, reported effect estimates with confidence intervals, and discrimination statistics) are reported in the article (Table 1, Table 2, Table 3 and Table 4 and Figure 2, Figure 3, Figure 4, Figure 5 and Figure 6) and Supplementary Materials. The complete data-extraction dataset and the R code used for the meta-analyses are available from the corresponding author upon reasonable request. The original data are available in the cited source publications.

## Supplementary Materials

The following supporting information can be downloaded at: https://www.mdpi.com/article/10.3390/healthcare14203543/s1, Table S1: Full database search strategies and targeted evidence update; Table S2: Full-text articles excluded and reasons for exclusion; Figure S1: Non-pooled hazard-ratio estimates for infection and short-term mortality; Figure S2: Twenty-eight-day mortality according to prevalent ACLF grade in severe alcohol-associated hepatitis; Figure S3: Study-reported adjusted associations of hepatic encephalopathy with short-term mortality; Figure S4: Sensitivity analysis of ACLF grade and time to death; Figure S5: Prognostic-score discrimination for short-term mortality in supportive populations; Figure S6: Short-term mortality stratified by ACLF diagnostic framework (post hoc analysis added in response to peer review). References [66,67,68,69,70,71,72,73,74,75,76,77,78,79,80,81,82,83,84,85,86,87] are cited in the Supplementary Materials.

## Abbreviations

Abbreviation	Definition
AARC	APASL ACLF Research Consortium
ACLF	Acute-on-chronic liver failure
AD	Acute decompensation
ALD	Alcohol-related liver disease
APASL	Asian Pacific Association for the Study of the Liver
AUROC	Area under the receiver operating characteristic curve
CI	Confidence interval
CLIF-C	Chronic Liver Failure Consortium
EASL-CLIF	European Association for the Study of the Liver–Chronic Liver Failure Consortium
HE	Hepatic encephalopathy
HR	Hazard ratio
ICU	Intensive care unit
MELD	Model for End-Stage Liver Disease
OR	Odds ratio
QUIPS	Quality In Prognosis Studies
REML	Restricted maximum likelihood
SOFA	Sequential Organ Failure Assessment
WGO	World Gastroenterology Organisation
