# Supplementary material for: Short-Term Mortality and Its Predictors in Alcohol-Related Acute-on-Chronic Liver Failure: A Systematic Review and Meta-Analysis
Source: Healthcare (Basel). 2026 Oct 21;14(20):3543. doi: 10.3390/healthcare14203543 (PMC13704466; doi:10.3390/healthcare14203543)
Supplement: Supplementary file 1 [file healthcare-14-03543-s001.zip › healthcare-4545773-supplementary.pdf]

Supplementary Materials

Short-Term Mortality and Its Predictors in Alcohol-Related Acute-on-Chronic Liver Failure: A Systematic Review and Meta-Analysis

Supplementary Table S1. Full database search strategies and targeted evidence update.

(A) Original search: sources, dates, yields, and exact database syntax.

Database	Last searched	Records retrieved
PubMed / MEDLINE	25 June 2026	128
Scopus	25 June 2026	99
Web of Science Core Collection	25 June 2026	43

All databases were searched from inception. Reference lists of included studies and relevant reviews were screened manually.

Exact strategy: PubMed / MEDLINE

(("acute-on-chronic liver failure"[Title/Abstract] OR "acute on chronic liver failure"[Title/Abstract] OR ACLF[Title/Abstract] OR "Acute-On-Chronic Liver Failure"[Mesh]) AND ("alcoholic liver disease"[Title/Abstract] OR "alcohol-related liver disease"[Title/Abstract] OR "alcohol-associated liver disease"[Title/Abstract] OR "alcoholic cirrhosis"[Title/Abstract] OR "alcohol-related cirrhosis"[Title/Abstract] OR "alcohol-associated cirrhosis"[Title/Abstract] OR "alcoholic hepatitis"[Title/Abstract] OR "alcohol-associated hepatitis"[Title/Abstract] OR "severe alcoholic hepatitis"[Title/Abstract]) AND (mortality[Title/Abstract] OR death[Title/Abstract] OR survival[Title/Abstract] OR outcome[Title/Abstract] OR prognosis[Title/Abstract] OR predictor*[Title/Abstract] OR "risk factor*"[Title/Abstract] OR "short-term mortality"[Title/Abstract] OR "28-day mortality"[Title/Abstract] OR "30-day mortality"[Title/Abstract] OR "90-day mortality"[Title/Abstract] OR "in-hospital mortality"[Title/Abstract]) AND (cohort[Title/Abstract] OR prospective[Title/Abstract] OR retrospective[Title/Abstract] OR observational[Title/Abstract] OR multicenter[Title/Abstract] OR "case-control"[Title/Abstract] OR "clinical study"[Title/Abstract])) NOT (transplant*[Title/Abstract] OR "liver transplantation"[Mesh]) NOT (animals[Mesh] NOT humans[Mesh])

Exact strategy: Scopus

TITLE-ABS-KEY("acute-on-chronic liver failure" OR "acute on chronic liver failure" OR ACLF) AND TITLE-ABS-KEY("alcoholic liver disease" OR "alcohol-related liver disease" OR "alcohol-associated liver disease" OR "alcoholic cirrhosis" OR "alcohol-related cirrhosis" OR "alcohol-associated cirrhosis" OR "alcoholic hepatitis" OR "alcohol-associated hepatitis" OR "severe alcoholic hepatitis") AND TITLE-ABS-KEY(mortality OR death OR survival OR outcome OR prognosis OR predictor* OR "risk factor*" OR "short-term mortality" OR "28-day mortality" OR "30-day mortality" OR "90-day mortality" OR "in-hospital mortality") AND TITLE-ABS-KEY(cohort OR prospective OR retrospective OR observational OR multicenter OR "case-control" OR "clinical study") AND NOT TITLE-ABS-KEY(transplant* OR "liver transplantation" OR "transplant recipient*") AND NOT TITLE-ABS-KEY(rat OR rats OR mouse OR mice OR animal*)

Exact strategy: Web of Science Core Collection

TS=("acute-on-chronic liver failure" OR "acute on chronic liver failure" OR ACLF) AND TS=("alcoholic liver disease" OR "alcohol-related liver disease" OR "alcohol-associated liver disease" OR "alcoholic cirrhosis" OR "alcohol-related cirrhosis" OR "alcohol-associated cirrhosis" OR "alcoholic hepatitis" OR "alcohol-associated hepatitis" OR "severe alcoholic hepatitis") AND TS=(mortality OR death OR survival OR outcome OR prognosis OR predictor* OR "risk factor*" OR "short-term mortality" OR "28-day mortality" OR "30-day mortality" OR "90-day mortality" OR "in-hospital mortality") AND TS=(cohort OR prospective OR retrospective OR observational OR multicenter OR "case-control" OR "clinical study") NOT TS=(transplant* OR "liver transplantation" OR "transplant recipient*") NOT TS=(rat OR rats OR mouse OR mice OR animal*)

(B) Revised comprehensive search: sources, dates, yields, and exact database syntax.

Database	Last searched	Records retrieved
PubMed / MEDLINE	27 September 2026	504
Scopus	28 September 2026	1022
Web of Science Core Collection	27 September 2026	535

All databases were searched from inception without language, date, or study-design restrictions. The revised search retrieved 2061 records; after removal of 886 duplicates, 1175 unique records were screened. Changes from the original strategy: the phrase-based alcohol block was replaced by truncated terms and MeSH headings; synonyms for ACLF and ACLF-specific scores (CLIF-C, CLIF-

SOFA, CLIF-OF, CANONIC, AARC score) were added; the outcome block was broadened with truncated terms; and the study-design block and the NOT transplant* restriction were removed. Only animal studies were excluded.

Exact revised strategy: PubMed / MEDLINE

("Acute-On-Chronic Liver Failure"[Mesh] OR "acute-on-chronic liver failure"[tiab] OR "acute on chronic liver failure"[tiab] OR "acute-on-chronic hepatic failure"[tiab] OR "acute on chronic hepatic failure"[tiab] OR ACLF[tiab] OR "CLIF-C"[tiab] OR "CLIF-SOFA"[tiab] OR "CLIF-OF"[tiab] OR CANONIC[tiab] OR "AARC score"[tiab]) AND ("Liver Diseases, Alcoholic"[Mesh] OR "Alcoholism"[Mesh] OR alcohol*[tiab] OR ethanol[tiab] OR ALD[tiab] OR ArLD[tiab] OR AALD[tiab]) AND ("Mortality"[Mesh] OR "Prognosis"[Mesh] OR "Survival Analysis"[Mesh] OR mortal*[tiab] OR death*[tiab] OR died[tiab] OR surviv*[tiab] OR fatal*[tiab] OR prognos*[tiab] OR predict*[tiab] OR outcome*[tiab] OR "risk factor*"[tiab] OR score*[tiab]) NOT (animals[Mesh] NOT humans[Mesh])

Exact revised strategy: Scopus

TITLE-ABS-KEY("acute-on-chronic liver failure" OR "acute on chronic liver failure" OR "acute-on-chronic hepatic failure" OR "acute on chronic hepatic failure" OR aclf OR "CLIF-C" OR "CLIF-SOFA" OR "CLIF-OF" OR canonic OR "AARC score") AND TITLE-ABS-KEY(alcohol* OR ethanol OR ald OR arld OR aald) AND TITLE-ABS-KEY(mortal* OR death* OR died OR surviv* OR fatal* OR prognos* OR predict* OR outcome* OR "risk factor*" OR score*) AND NOT TITLE-ABS-KEY(rat OR rats OR mouse OR mice OR murine)

Exact revised strategy: Web of Science Core Collection

TS=("acute-on-chronic liver failure" OR "acute on chronic liver failure" OR "acute-on-chronic hepatic failure" OR "acute on chronic hepatic failure" OR ACLF OR "CLIF-C" OR "CLIF-SOFA" OR "CLIF-OF" OR CANONIC OR "AARC score") AND TS=(alcohol* OR ethanol OR ALD OR ArLD OR AALD) AND TS=(mortal* OR death* OR died OR surviv* OR fatal* OR prognos* OR predict* OR outcome* OR "risk factor*" OR score*) NOT TS=(rat OR rats OR mouse OR mice OR murine)

(C) Targeted evidence update and cohort linkage (including potentially overlapping reports identified by the revised search).

Report	Eligibility/cohort status	Treatment in the review
Saini et al., 2025 [25]	Eligible independent cohort	Included as an independent cohort and in eligible endpoint-specific mortality analyses.
Glisic et al., 2026 [26]	Eligible potentially overlapping secondary report	Narrative only; not counted as an independent cohort or pooled.
Maiwall et al., 2023 [52]	Eligible potentially overlapping secondary report	Narrative only; not counted as an independent cohort or pooled.
Kumar et al., 2026 [50]	Eligible potentially overlapping secondary report	Narrative only; not counted as an independent cohort or pooled.
Thanapirom et al., 2022 [61]	Eligible potentially overlapping secondary report	Narrative only; not counted as an independent cohort or pooled.
Shalimar et al., 2017 [59]	Eligible potentially overlapping secondary report	Narrative only; not counted as an independent cohort or pooled.

Note: The Glisic report [26] originated from the same Serbian center and shared investigators with Matovic Zaric et al. [16]; participant-level overlap could not be confirmed. It was therefore used only for contextual interpretation and was not counted or pooled as an independent cohort. Maiwall et al. [52], Kumar et al. [50], and Thanapirom et al. [61] derive from the APASL ACLF Research Consortium registry and potentially overlap with Duseja et al. [9]; Shalimar et al. (2017) [59] was conducted at the same center and during an overlapping period as Sonika et al. [60]. These reports were likewise used only for contextual interpretation.

Supplementary Table S2. Full-text articles excluded and reasons for exclusion (rows 13–17: reports excluded after the revised search)

No.	Author, year	Article title	Reason for exclusion
1	Agrawal et al., 2015 [66]	Simple organ failure count versus CANONIC grading system for predicting mortality in acute-on-chronic liver failure	Mixed ACLF cohort; alcohol was common, but no separate alcohol-specific ACLF predictor data for meta-analysis.
2	Dhiman et al., 2014 [67]	Chronic Liver Failure-Sequential Organ Failure Assessment is better than the Asia-Pacific Association for the Study of Liver criteria for defining acute-on-chronic liver failure and predicting outcome	Mixed cirrhosis/ACLF cohort; alcohol common, but no separate extractable alcohol-related ACLF effect size.
3	Duseja et al., 2010 [68]	Non-hepatic insults are common acute precipitants in patients with acute on chronic liver failure (ACLF)	Mixed ACLF etiologies and precipitants; alcohol subgroup not separately extractable for mortality predictors.
4	Duseja et al., 2013 [69]	APACHE II score is superior to SOFA, CTP and MELD in predicting the short-term mortality in patients with acute-on-chronic liver failure (ACLF)	Mixed ACLF cohort; mostly alcohol-related but not restricted to alcohol-related ACLF; limited extractable alcohol-specific predictor data.
5	Garg et al., 2012 [70]	Clinical profile and predictors of mortality in patients of acute-on-chronic liver failure	Mixed etiology ACLF; alcohol was only one subgroup, without separate alcohol-specific predictor estimates.
6	Khot et al., 2014 [71]	Prognostic factors in acute-on-chronic liver failure: a prospective study from western India	Mixed ACLF cohort; hepatitis B and cryptogenic disease were common; alcohol-specific mortality predictors not separately reported.

7	Mikolasevic et al., 2015 [72]	Clinical profile, natural history, and predictors of mortality in patients with acute-on-chronic liver failure (ACLF)	Mixed ACLF cohort; alcohol was the most common underlying disease, but separate alcohol-specific effect sizes were not extractable.
8	Gupta et al., 2017 [73]	Impact of Hepatic and Extrahepatic Insults on the Outcome of Acute-on-Chronic Liver Failure	Mixed ACLF population; alcohol was common, but results were stratified by hepatic/extrahepatic insult rather than alcohol-related ACLF.
9	Halder et al., 2023 [74]	Clinical Profile and Prognostic Markers of Acute on Chronic Liver Failure (ACLF): A Single-center Experience from East India	Mixed ACLF cohort; alcohol was an etiology/acute insult subgroup, but alcohol-specific mortality predictors were not separately extractable.
10	Khatua et al., 2023 [75]	Acute Kidney Injury (AKI) at Admission Predicts Mortality in Patients With Severe Alcoholic Hepatitis (SAH)	Severe alcoholic hepatitis cohort; ACLF was reported as a complication/prevalence, but the study was not an ACLF-specific mortality-predictor cohort.
11	Bernsmeier et al., 2020 [76]	Leucocyte ratios are biomarkers of mortality in patients with acute decompensation of cirrhosis and acute-on-chronic liver failure	Mixed acute decompensation/ACLF cohort; alcohol subgroup mentioned, but not a dedicated alcohol-related ACLF cohort.
12	Bruns et al., 2017 [77]	Low serum transferrin correlates with acute-on-chronic organ failure and indicates short-term mortality in decompensated cirrhosis	Decompensated cirrhosis/ACLF-risk cohort; not restricted to alcohol-related ACLF and no separate alcohol-specific ACLF predictor extraction.
13	Cold et al., 2019 [78]	Sepsis-related organ failure assessment score is a strong predictor of survival in acute-on-chronic liver failure	ICU cohort of decompensated alcohol-related cirrhosis; ACLF was not defined by any recognized diagnostic criteria (not an ACLF population).
14	Kośnik et al., 2024 [79]	Role of single-pass albumin dialysis in acute-on-chronic liver failure of alcoholic origin: a single-center experience	Intervention-selected cohort (all patients received single-pass albumin dialysis); no prognostic analysis applicable to an unselected alcohol-related ACLF population.
15	Nidhin et al., 2025 [80]	Performance of five prognostic scores in predicting short-term mortality in patients with acute-on-chronic liver failure	Mixed-etiology ACLF cohort; alcohol-related ACLF was not analyzed separately (alcohol reported only as a baseline characteristic).
16	Shalimar et al., 2016 [81]	Acute on chronic liver failure because of acute hepatic insults: Etiologies, course, extrahepatic organ failure and predictors of mortality	Duplicate cohort from the same center and period as the included reports of Shalimar et al., 2017 [59] and Sonika et al., 2018 [60]; mixed etiology.
17	Singal et al., 2020 [82]	Increasing burden of acute-on-chronic liver failure among alcohol-associated liver disease in the young population in the United States	Administrative database (National Inpatient Sample): hospitalization-level data, ACLF identified from diagnosis codes, and in-hospital mortality only.

Note: Exclusion reasons are reported at the report level and reflect the absence of a separately extractable alcohol-related ACLF analysis under the final eligibility rules. Mixed cohorts were retained only when the alcohol-related subgroup or a prespecified supportive role was sufficiently clear; otherwise, they were excluded to avoid nonseparable indirect evidence.

Reports sought for retrieval but not retrieved after the revised search (n = 5): Chauhan et al., 2020 [83] (J. Assoc. Physicians India); Fakhoury et al., 2026 [84] (Hepatol. Commun.); Guo et al., 2023 [85] (J. Clin. Hepatol., in Chinese); Hui et al., 2025 [86] (J. Clin. Hepatol., in Chinese); and Zhu et al., 2021 [87] (J. Clin. Hepatol., in Chinese). These reports are shown as “Reports not retrieved” in the PRISMA flow diagram (main-text Figure 1).

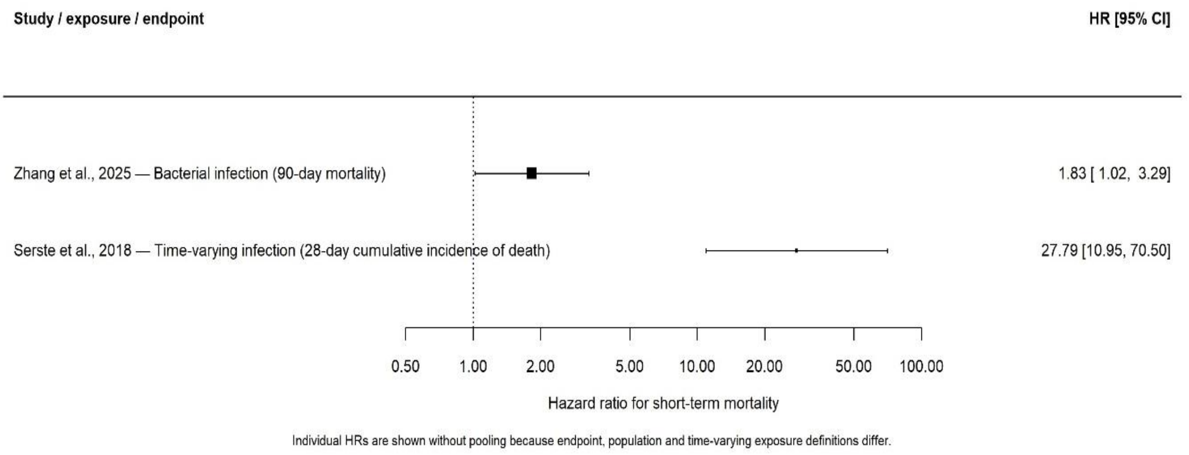

Supplementary Figure S1. Non-pooled hazard-ratio estimates for infection and short-term mortality. Zhang et al. [24] evaluated baseline bacterial infection and 90-day mortality in alcohol-related ACLF, whereas Sersté et al. [19] evaluated time-varying infection and 28-day cumulative incidence of death in severe alcohol-associated hepatitis. No pooled estimate was calculated because exposure timing, analysis populations, and mortality endpoints differed.

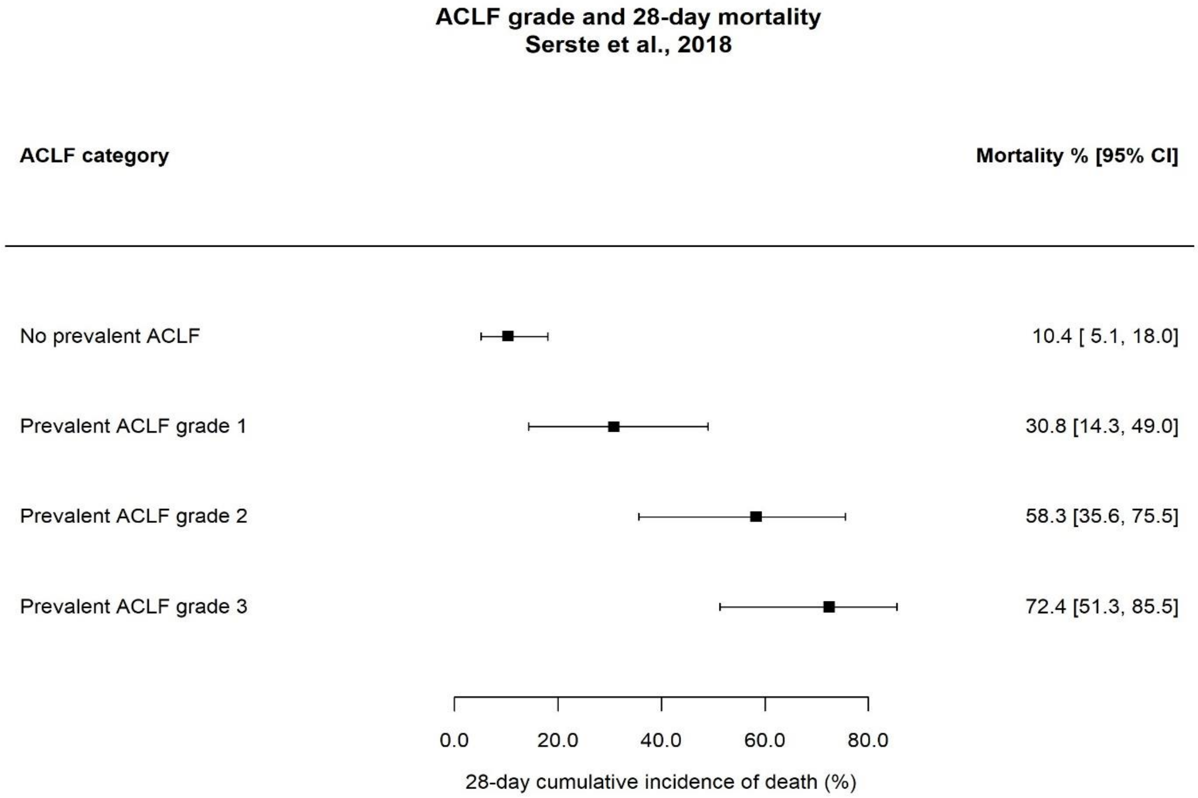

Supplementary Figure S2. Twenty-eight-day mortality according to prevalent ACLF grade in severe alcohol-associated hepatitis. Cumulative incidence increased progressively from no prevalent ACLF to grades 1, 2, and 3. Liver transplantation was treated as a competing event.

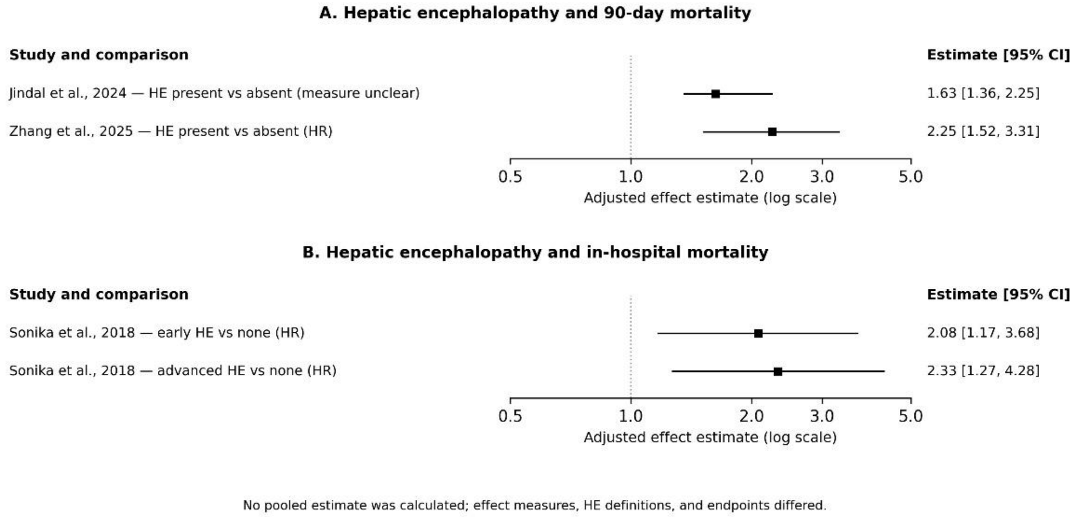

Supplementary Figure S3. Study-reported adjusted associations of hepatic encephalopathy with short-term mortality. (A) 90-day mortality. (B) In-hospital mortality (Sonika et al. [60], added from the revised search). Estimates are presented separately because effect measures, definitions and timing of hepatic encephalopathy, and endpoints differed. For Jindal et al. [11], the confidence interval reported in Table 3 of the source article (1.36–2.25) was used; the Results text reports 1.36–2.35, and the effect measure was inconsistently labeled. No pooled estimate was calculated. CI, confidence interval; HE, hepatic encephalopathy; HR, hazard ratio.

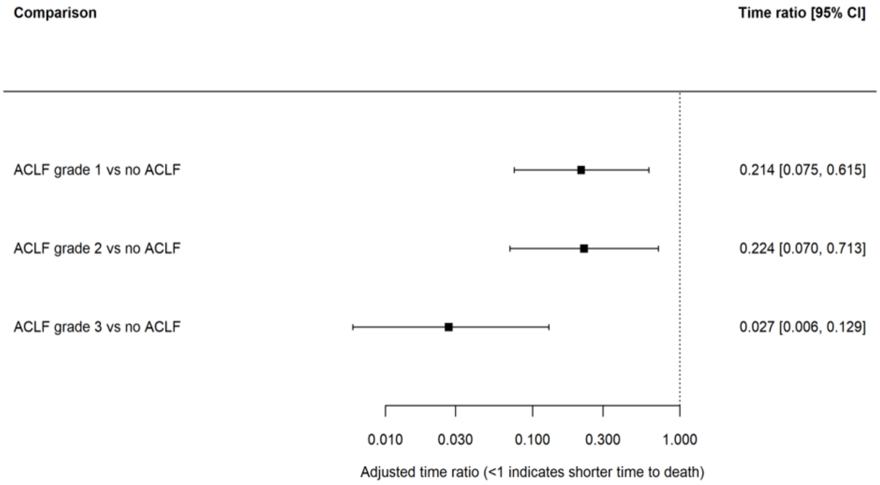

Supplementary Figure S4. Sensitivity analysis of ACLF grade and time to death. Adjusted time ratios were derived from an accelerated failure-time model in a mixed-etiology decompensated-cirrhosis cohort. Values below 1 indicate shorter time to death relative to patients without ACLF; these estimates were not combined with fixed-time mortality effects.

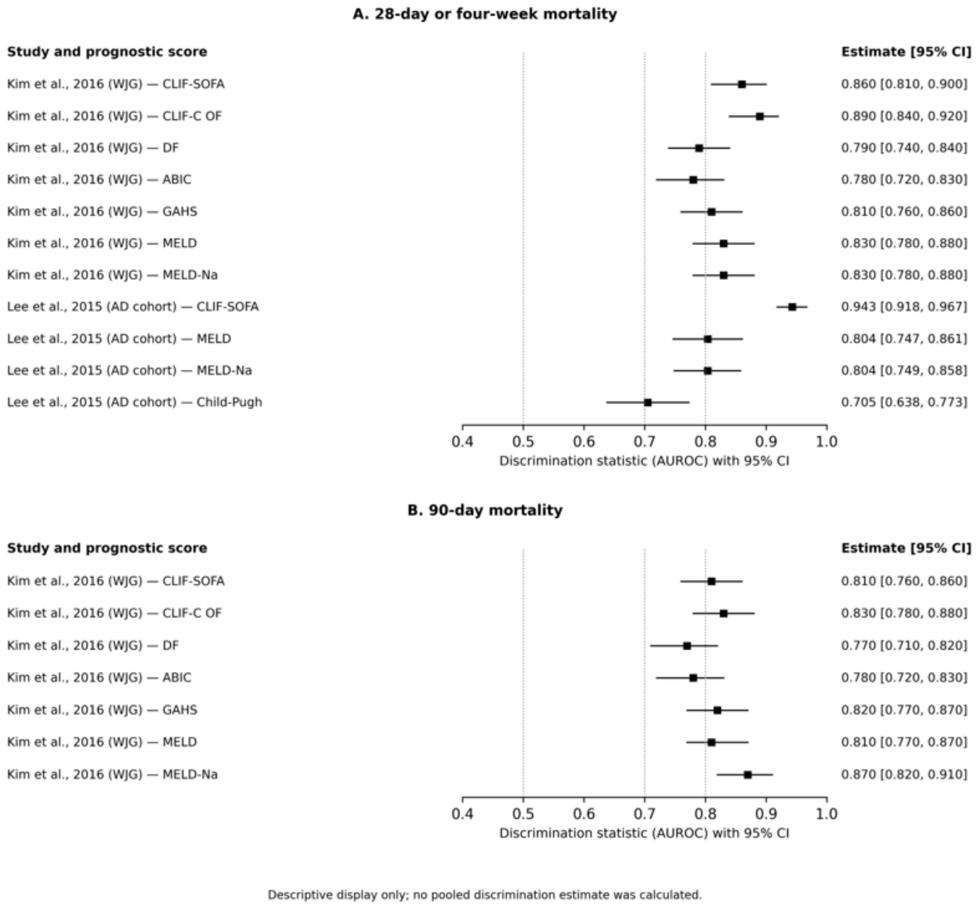

Supplementary Figure S5. Prognostic-score discrimination for short-term mortality in supportive populations. (A) 28-day or four-week mortality in severe alcohol-associated hepatitis (Kim et al., 2016, WJG [14]) and in acutely decompensated alcohol-related cirrhosis including patients without ACLF (Lee et al., 2015 [51]; whole cohort, n = 345). (B) 90-day mortality in severe alcohol-associated hepatitis (Kim et al., 2016, WJG [14]). These estimates were not restricted to a separately analyzed ACLF subgroup; therefore, no pooled discrimination estimate was calculated. Two further supportive severe alcohol-associated hepatitis cohorts reported discrimination without confidence intervals: Rutledge et al. [58] (AUROC 0.73 for CLIF-C ACLF, 0.73 for MELD-Age, 0.72 for ACLF-Age, 0.62 for Maddrey discriminant function, and 0.63 for Lille) and Perricone et al. [56] (C-index for death or liver transplantation 0.708 for MELD-Na, 0.702 for prevalent ACLF 2–3, and 0.691 for CLIF-C ACLF).

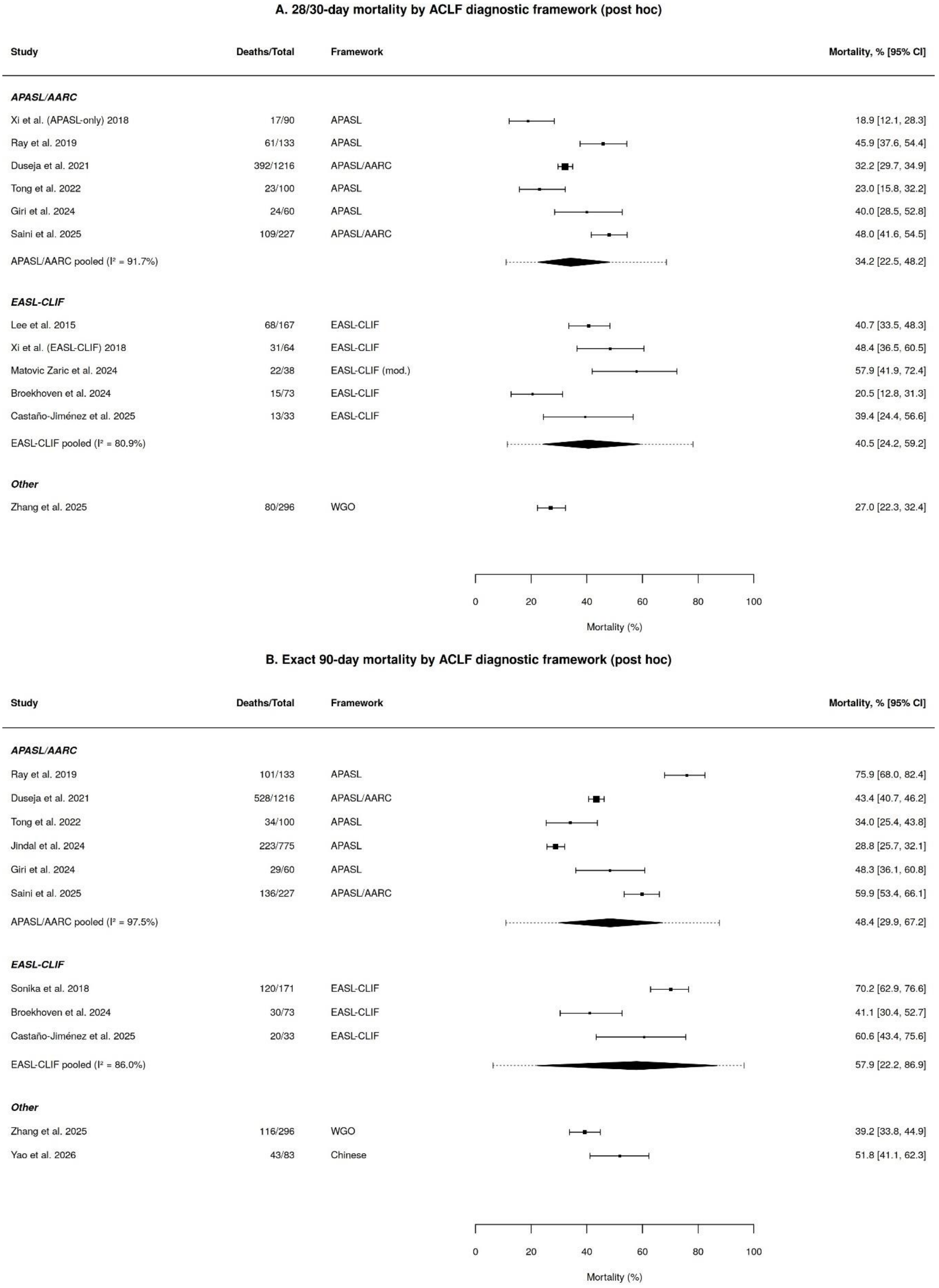

Supplementary Figure S6. Short-term mortality stratified by ACLF diagnostic framework (post hoc analysis added in response to peer review). (A) 28/30-day mortality. (B) Exact 90-day mortality. Random-effects pooled estimates (REML on the logit scale, Hartung–Knapp confidence intervals, dotted lines showing 95% prediction intervals) are shown for strata with at least two cohorts; the cohort of Xi et al. [62] contributed its APASL-only and EASL-CLIF subgroups to the respective strata. Single-cohort strata (WGO; Chinese criteria) are shown descriptively. Differences between APASL/AARC- and EASL-CLIF-defined cohorts were not statistically significant ($p = 0.46$ at 28/30 days; $p = 0.48$ at 90 days).
